# Histone Deacetylase Inhibition Enhances Tissue Plasminogen Activator Release Capacity in Atherosclerotic Man

**DOI:** 10.1371/journal.pone.0121196

**Published:** 2015-03-25

**Authors:** Kristina Svennerholm, Michael Haney, Björn Biber, Erik Ulfhammer, Ott Saluveer, Pia Larsson, Elmir Omerovic, Sverker Jern, Niklas Bergh

**Affiliations:** 1 Anesthesiology and Intensive Care Medicine, Institute of Clinical Science, Sahlgrenska Academy, University of Gothenburg, Gothenburg, Sweden; 2 The Wallenberg Laboratory for Cardiovascular Research, Institute of Medicine, Sahlgrenska Academy, University of Gothenburg, Gothenburg, Sweden; 3 Anesthesiology and Intensive Care Medicine, Institute for Surgical and Perioperative Science, Umeå University, Umeå, Sweden; Maastricht University Medical Center, NETHERLANDS

## Abstract

The expression of the tissue plasminogen activator (t-PA) gene appears to be under epigenetic control and can be affected by histone deacetylation inhibition. The study aimed to test if histone deacetalyase inhibitor treatment lead to increased t-PA release or reduced exhaustion in t-PA release in response to stimulation, as well as change in plasminogen activator inhibitor-1 (PAI-1) in subjects with coronary disease. In this clinical study, 16 post-myocardial infarction subjects, the perfused forearm model was used with isoprenaline provocation during 20 minutes, to stimulate local t-PA release. Each subject was measured twice on the same day (repeated stimuli sequences) as well as on two different occasions, without treatment and after four weeks of treatment with valproic acid (500 mg, twice daily). Net forearm release for t-PA in response to isoprenaline at minutes 1.5, 3, 6, 9, 12, 15 and 18 was measured, allowing assessment of cumulative t-PA release. There was a reduction in the exhaustion of cumulative t-PA release during repeated and prolonged stimulation with valproic acid treatment compared to non-treatment. Plasma PAI-1 antigen was decreased following treatment compared to non-treatment (18.4 ± 10.0 vs. 11.0 ± 7.1 nanograms/ml respectively, mean with 95% confidence interval). These findings demonstrate that histone deacetylation inhibition increases the capacity for endogenous t-PA release in subjects with vascular disease. Furthermore, the fibrinolytic balance is favored with suppressed PAI-1 levels. More studies are needed to establish the clinical relevance of these findings.

**Trial registration:**

EU Clinical Trials Register 2012-004950-27

## Introduction

In health, normal endothelial function is important for preventing thrombus formation [1]. For patients with a thrombotic tendency or deficiencies in own fibrinolytic activity, thrombosis can be a life-threatening problem [2–4]. Improvement in endogenous fibrinolytic activity could be protective and therapeutic for these patient groups. To date, different anti-thrombotic treatments may be employed, targeting platelets or the coagulation cascade, but there is no therapy available to increase endogenous fibrinolytic capacity.

Tissue plasminogen activator (t-PA) is the key enzyme regulating fibrinolysis. Functional t-PA activity is the result of the plasma balance between t-PA and its inhibitor, plasminogen activator inhibitor-1 (PAI-1). Interpretation of t-PA levels should always be done in relation to PAI-1 levels [5–7]. Healthy endothelium is the main source of endogenous t-PA. Most of the synthesized t-PA is stored within the endothelial cells in small vesicles while only a small portion is constitutively released into the vascular lumen. Facultative t-PA release from the intracellular storage pools occurs in response to local stimuli, for example during local injury, rupture of an atherosclerotic plaque, or at the start of local thrombus formation. It is the initial rapid t-PA release, during the first minutes, which is most important in preventing a thrombus from becoming established [8,9]. Circulating t-PA in health is short-lived in the bloodstream, where the largest part goes into complex with PAI-1 and is inactivated, and later is eliminated through hepatic clearance [10].

The fibrinolytic response has been shown to be deficient in large patient groups with cardiovascular risk factors such as hypertension, coronary disease, active smokers, renal failure as well as obesity [11–13]. Currently, the only way of regulating the plasma-levels of t-PA is by systemic administration of recombinant protein, an established treatment alternative in acute myocardial infarction, stroke and pulmonary embolism. This intervention confers risk for severe bleeding complications, since large systemic amounts of t-PA are needed to achieve local effects. Since exogenous t-PA is given late in the thrombus stabilization process, the effect of a given amount is much less compared to endogenous t-PA which is present before and during thrombus generation [8,9]. Hence, stimulation of endogenous production and release of t-PA is a superior approach for improving fibrinolytic response. This can be achieved by increasing the endothelial capacity of t-PA production, storage, release and/or by altering PAI-1 levels.

It has been demonstrated that t-PA expression can be altered by epigenetic mechanisms related to degree of DNA histone acetylation in cultured human endothelial cells [14–17]. Valproic acid (VPA), a histone deacetylase inhibitor (HDACi), has been demonstrated to increase t-PA expression not only in *in vitro*, but also in an in vivo large animal model, by our group [18]. In the preceeding related clinical study by our group, examining VPA effect in healthy volunteers, VPA treatment was shown to lower plasma PAI-1 antigen levels, with a profibrinolytic effect on t-PA/PAI-1 ratio [19].

In this study, we aimed to test HDACi effects on t-PA expression in a clinical cohort where t-PA production and release capacity is predicted to be decreased. We hypothesized that VPA treatment would lead to increased storage pools of t-PA within endothelial cells which would be manifest as increased t-PA release capacity upon stimulation; that VPA treatment reduces exhaustion in t-PA release upon restimulation, as well as a decrease in PAI-1 plasma concentrations. We tested this in post-myocardial infarction subjects using the perfused forearm model with isoprenaline (ISP) stimulation, without and with VPA treatment.

## Material and Methods

### Subjects

The protocol for this trial and supporting TREND checklist are available as supporting information; see S1 Checklist and S1 Protocol. The study was approved by the regional Human Research Ethical Board at the University of Gothenburg (Dnr 935-12, 20130206) and by the Medical Product Agency in Sweden (EudraCT nr: 2012-004950-27, registered at https://www.clinicaltrialsregister.eu/ctr-search/trial/2012-004950-27/SE), and conforms to the Declaration of Helsinki. Informed consent was obtained from all subjects prior to their participation. Potential subjects were identified from the Swedish national coronary angiographic and angioplasty register (Riks-HIA/SCAAR http://www.ucr.uu.se/scaar/index.php), where all patients meeting the criteria were invited to participate in the study. Inclusion criteria were the following: male adult patients aged less than 85 years, treated for myocardial infarction at least one year ago at the Sahlgrenska University Hospital, Gothenburg, Sweden. The exclusion criteria were the following: active smoking, body mass index > 35 kg/m^2^, symptomatic coronary syndrome, heart failure or uncontrolled hypertension, anticoagulation therapy other than ASA, medications interacting with VPA, chronic diseases contraindicating VPA treatment, known malignancy, active infection, psychiatric disorder, alcoholism, epilepsy, or inability to understand study information. Subjects meeting these criteria where invited by mail to participate in the study. Those who responded positively were invited to undergo a basic physical examination, ECG and a standardized set of ‘inclusion’ blood tests including blood cell counts, electrolytes, creatinine, liver enzymes, coagulation tests, glucose, and high sensitive C-reactive protein (hs-CRP). Detailed study information was provided and written informed consent was obtained.

### Study design

**Fig 1:**
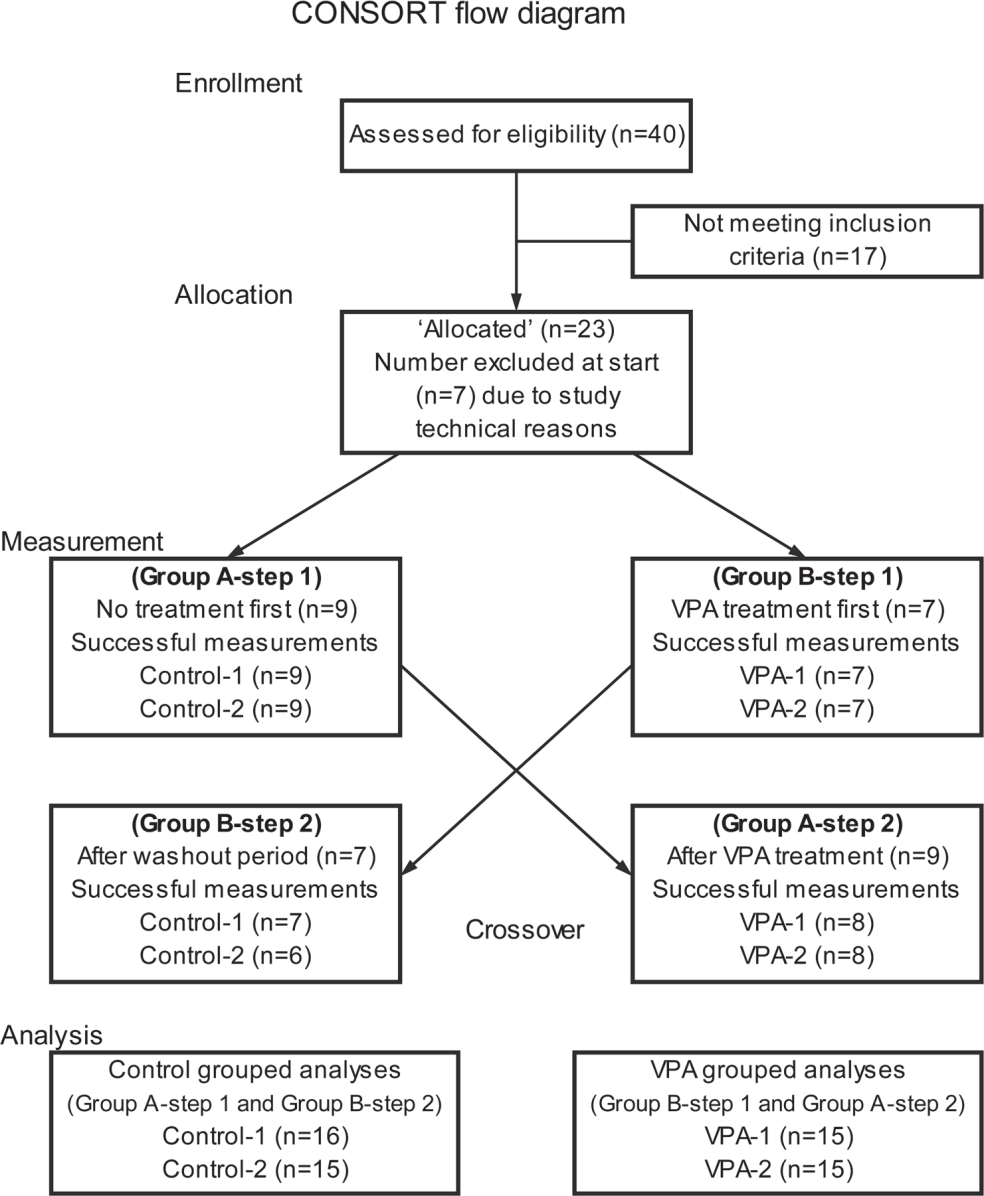
CONSORT subject flow diagram. Subjects were placed initially in one of two groups, where one group was treated for 4 weeks with VPA before being measured, and the other group was measured directly as Controls. After the first measurement, the VPA treated group rested for 4 weeks with a washout period before being measured again Control. The subjects that first were Controls, were measured again after 4 weeks of VPA treatment. The subjects first treated with VPA were measured and then observed a 4 week washout and rest period before they were studied again as Controls. All subjects measured with VPA were combined for grouped analysis (same for Control measurements).

This was a prospective study in a selected cohort where treatment effects of VPA on stimulated t-PA release and static PAI-1 levels were measured. All subjects received valproic acid (Ergenyl Retard, Sanofi Aventis, 500 mg, twice daily) for four weeks before the stimulated t-PA release measurements. Four weeks was chosen as a treatment interval in order to balance both a VPA HDACi effect with equilibrium as well as a matching non-treatment time interval where VPA effect resolution could be presumed [20]. All subjects underwent stimulated t-PA release measurement on two separate days: one with and one without VPA treatment. At each measurement day, there were two identical sequences, with one hour rest in between in order to assess exhaustion in the stimulated t-PA release pattern. Half the subjects (randomly assigned by lot) had their non-treatment study first, and then received four week VPA treatment before the second measurements. The other subjects had their four week VPA treatment before the first measurements and then a four week wash-out period before the second measurements (Fig. 1). This crossing-over was included in the study design in order to try to minimize possible effects of novelty and subject anxiety on the stimulated t-PA release measurements.

### Measurement of stimulated t-PA release

On the test days, subjects were fasting, and were restricted from theophylline- or caffeine containing foods for the previous 12 hours. They were also restricted from non-steroid anti-inflammatory drugs, alcohol, and strong physical activity for the preceding 24 hours, as well as vitamin substitution for the preceding ten days. Subjects were instructed to take their usual medications, and VPA (if last day of treatment) at 7 AM on the test days. The tests were conducted in a quiet room, with subjects resting in a reclining position.

Total stimulated forearm t-PA release was measured in a well-established forearm arterial and venous cannulation and plethysmographic model [21–22]. Briefly, this included measurement of the circumference of the non-dominant forearm, and its volume as determined by water displacement. A 4 French arterial catheter (Careflow, Becton Dickinson) in the brachial artery of the non-dominant arm was used for drug infusion, arterial blood sampling, and pressure monitoring. For venous sampling, a short cannula was introduced retrograde into an antecubital vein in the ipsilateral arm. On test days, the same set of blood samples as at ‘inclusion’ were collected as well as for plasma-cholesterol, lipoproteins, fasting glucose and VPA. Arterial and venous blood capillary tube spun haematocrits (Hct) were measured.

To assess forearm blood flow (FBF), expressed in mL • min^-1^ • 100 ml^-1^ tissue, venous occlusion plethysmography was performed. After each venous blood sample, mean FBF was calculated from three to five separate venous occlusion sequences, using commercial software (MAPPC, Elektromedicin AB; Kullavik, Sweden). Forearm t-PA release was measured before, during and after an intra-arterial infusion of isoprenaline (ISP) (Hospira, Lake Forest, USA), which was dosed as follows: 300 nanograms • ml^-1^ in isotonic saline, at a constant rate of 1 ml • min^-1^ over 20 minutes. Isoprenaline was chosen when the initial study design stimulus drug, substance P, was no longer commercially available. One set of arterial and venous blood for PAI-1 analysis was taken before stimulation, since it is recognized that PAI-1 is not released by forearm stimulation [21]. Arterial and venous baseline samples for t-PA were collected in triplicate both pre- and post-infusion. No arterial blood samples were obtained during the ISP infusion phase, when venous samples were collected at 1.5, 3, 6, 9, 12, 15, and 18 minutes. Two ml of blood were collected using chilled syringes, and placed in chilled glass test tubes, each containing 0.2 ml 0.45 M sodium citrate buffer, pH 4.3. The samples were then centrifuged for 20 min at 2000 G and 4°C. Plasma was stored at −70°C in two aliquots in sealed plastic vials until being thawed for analysis.

Enzyme-linked immunosorbent assays (ELISA) were used for quantitative determination of PAI-1 (Technozym PAI-1ELISA, Haemochrom Diagnostica, Vienna, Austria) and total t-PA antigen (TriniLize t-PA antigen ELISA, Trinity Biotech, Bray, Ireland) in plasma. Plasminogen activator inhibitor-1 was calculated as the mean of arterial and vein samples taken during the pre-ISP period. Each sample for t-PA was assayed in duplicate and mean concentration was used for analysis. All inclusion blood tests and plasma VPA concentrations were analyzed at the Department of Clinical Chemistry, Sahlgrenska University Hospital, Gothenburg, Sweden.

### t-PA release

The basal release and the net release/uptake of total t-PA from the forearm vascular bed were calculated as the product of the arterio-venous concentration difference of t-PA (C_V_ – C_A_) and the local plasma flow (FBF x (101-Hct)/100), using the following formula:

(C_V_ – C_A_) x FBF x (101-Hct)/100 where C_V_ is the venous concentration of t-PA, and C_A_ is the arterial concentration of t-PA.

### Statistics

In designing the study, considerations for study power and sampling size included an estimated 30% increase in t-PA release with VPA treatment along with a within-group variability of 50% of the total response in a another report [23], power of 0.8 and an α value of 0.05, which provided an estimated sample size of 22.

Descriptive statistics for demographic variables were presented with mean values ± standard deviation. In figures, variation with the group at a specific time point is presented with a 95% confidence interval (CI). The t-PA measurements were grouped for control and VPA measurements, and a paired t-test was performed where each subject provided their own control measurement. Testing for changes in t-PA during the ISP stimulation sequence was performed with repeated measures ANOVA. Area under the curve (AUC) was calculated as the integral of the t-PA release point measurements and time during the ISP sequence, and this represented cumulative t-PA release during stimulation. Cumulative t-PA release was compared between grouped measurements using AUC at specific time points and paired t-testing. For assessment of exhaustion between the first and second ISP sequences (same day), AUC measures were combined as a ratio, using the first measure for each individual as 100%, and then reporting the second measure as a percent of the first at each time point during the ISP sequence. These values were grouped, and compared using paired t testing. A significant difference was noted in testing when the p value was less than 0.05.

## Results

### Subjects

Subjects were recruited and studied during 2013. Eighty potential subjects received a letter inviting participation. Interviews were conducted with 40 who responded, where exclusion criteria eliminated 17, and 23 subjects were included in the study. Seven subjects did not successfully complete the study protocol due to different reasons, including difficulty with vein or artery cannulation, gastroenteritis and vaso-vagal reaction during measurement, leaving 16 subjects who completed the study protocol (Fig. 1) (Table 1).

**Table 1:** Demographic data.

Age (years)	70 ± 8.7
Body mass index (kg/m^2^)	28.3 ± 3.0
Heart rate (bpm)	62 ± 8
Systolic blood pressure (mm Hg)	138 ± 9
Diastolic blood pressure (mm Hg)	81 ± 9
Mean arterial pressure (mm Hg)	100 ± 11
Hemaglobin (g/L)	150 ± 9
Creatinine (μmol/L)	86 ± 15
Glucose (mmol/L)	6.2 ± 1.0
Cholesterol (mmol/L)	3.85 ± 0.62
Triglycerides (mmol/L)	1.13 ± 0.44
Low-density lipoprotein cholesterol (mmol/L)	2.42 ± 0.50

Abbreviations: kg = kilogram, m^2^ = square meter, bpm = beats per minute, mm Hg = millimeters of mercury, g = gram, L = liter, μmol = micromole, mmol = millimole. Results shown as mean ± SD, (n = 16).

The VPA treatment led to average VPA plasma concentrations in the therapeutic (anti-seizure) range (300–700 μmol/L) (Table 2). Only one subject had blood VPA concentration below therapeutic range (270 μmol/L). There was a decrease in PAI-1 concentration with VPA treatment (18.4 ± 10.0 vs. 11.0 ± 7.1 nanograms/ml respectively, p = 0.01). Concerning coagulation and inflammation, VPA treatment was associated with small decreases in mean platelet count and fibrinogen concentration, as well as a small increase in mean PT-INR, and a small decrease in mean hs-CRP (Table 2). During VPA treatment, one subject had total white cell blood count below normal limits (3.3 • 10^9^ g/L), three subjects had platelet counts that decreased to between 120 and 150 • 10^9^ g/L.

**Table 2:** Coagulation and inflammation.

	Inclusion	After VPA	p =
Valproic acid (mmol/L)	non detectable	438 ± 44	
Thrombocytes (x10^9^·g/L)	235 ± 25	190 ± 20	< 0.001
D-dimer (mg/L)	0.68 ± 0.22	0.66 ± 0.25	0.924
Fibrinogen (g/L)	3.4 ± 0.3	2.67 ± 0.30	< 0.001
Activated partial thromboplastin time (s)	36.2 ± 2.0	49.3 ± 23.2	0.254
Prothrombin time-INR	1.04 ± 0.04	1.09 ± 0.03	0.014
High-sensitivity C-reactive protein (mg/L)	1.85 ± 1.16	1.35 ± 0.84	0.04
Plasminogen activator inhibitor-1 (ng/ml)	19.6 ± 10.0	11.0 ± 7.1	0.01

Abbreviations: CI = confidence interval, μmol = micromole, g = gram, L = liter, mg = milligram, s = second, ng = nanogram, ml = milliliter. Comparisons made with a paired t test. Data shown as mean with 95% CI, (n = 16).

### Grouped results during stimulated t-PA release sequences

On the study days all subjects were stimulated for acute t-PA release twice during the same day, without VPA treatment (Control-1 and Control-2) and, with VPA treatment (VPA-1 and VPA-2). Since the washout period was very long, and HDACi effects presumed long extinguished, the grouped measures for Controls were combined into one group for analysis, and all the measurements with VPA treatment were combined (Fig. 1). Mean arterial blood pressure and heart rate were not different between control and VPA measurements during 20 minutes of ISP infusion (data not shown). Infusion of ISP resulted in rapid increases in FBF, from approximately 4 ml/min/100ml to approximately triple that rate which was sustained throughout the ISP infusion, with no differences between first and second measurement on the same day, as well as no observed difference between Control and VPA grouped measurements (results not shown).

**Fig 2:**
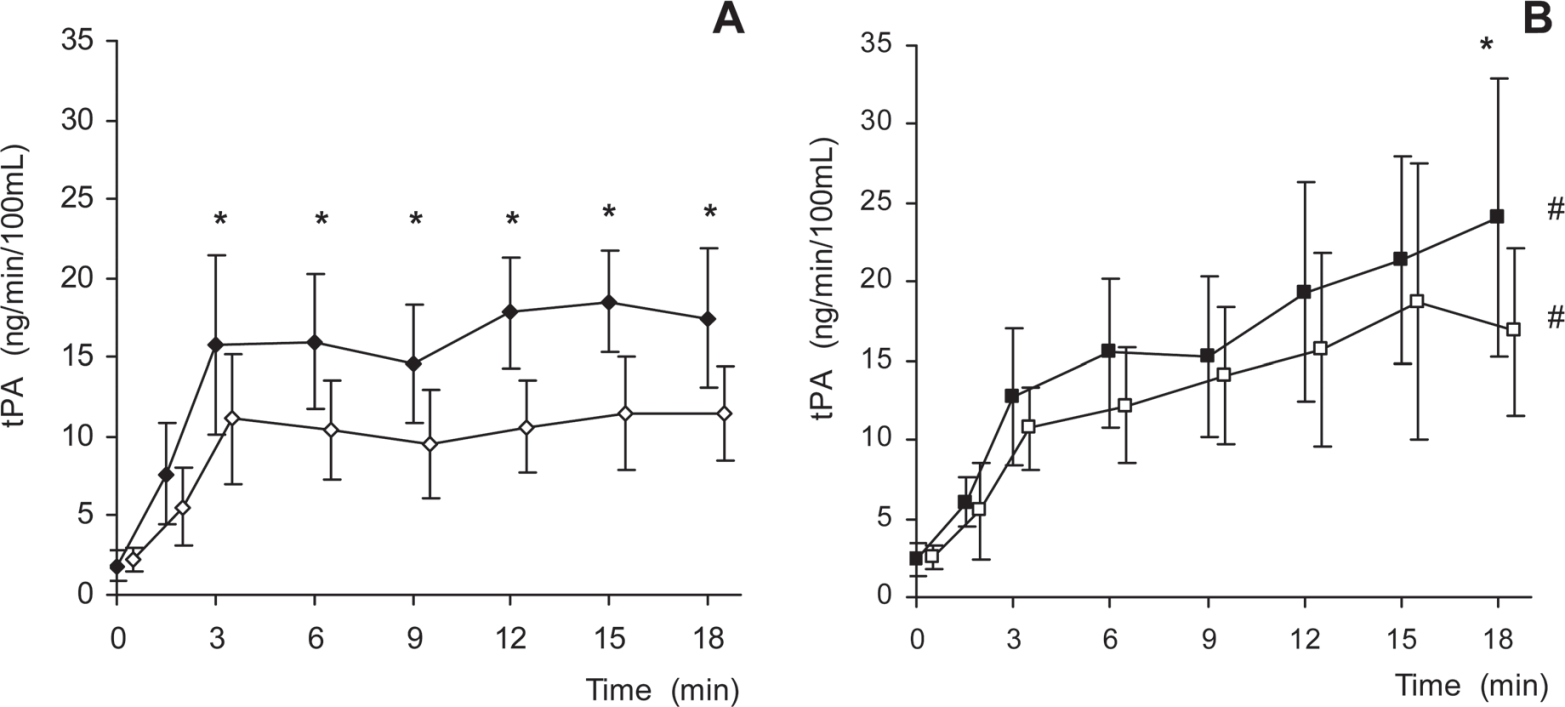
Forearm t-PA net release on the same day, first and second measurements. Panel A demonstrates non-treatment grouped t-PA fluxes during ISP stimulation for the first measurement sequence (Control-1, filled diamond, n = 16), and second (Control-2, open diamond, n = 15). Panel B shows the VPA treated grouped t-PA fluxes for the first measurement sequence (VPA-1, filled square, n = 15) and second (VPA-2, open square, n = 15). First, there are linear and progressive increases in t-PA release during the course of the ISP stimulation in the VPA group (Panel B), but not in the Control group (Panel A) It is notable that for Control, there are lower t-PA fluxes at each time point (3–18 minutes) during the second (same day) stimulation, but that this is not the case for the VPA grouped measures, until 18 minutes of stimulation. There is clear lower level in the response for the second stimulation (one hour later) for Controls that is not observed with VPA treatment. Data are presented as mean with 95% confidence intervals. # indicates p value less than 0.05 using repeated measured ANOVA within groups for change in values during the interval between 3–18 minutes. * indicates p value less than 0.05 using repeated paired t tests.

For net t-PA release at different time points during the ISP provocation several distinct patterns can be observed. First, before ISP was infused, baseline observations show that VPA treatment had no effect on grouped baseline t-PA flux levels (Fig. 2). Infusion of ISP led to a manifold increase in forearm t-PA release starting at three minutes, and which was sustained over the whole ISP infusion period. One notable finding is that for the control grouped measurements from minutes 3 to 18, the responses were consistent during the course of first and second measurement sequences (repeated measured ANOVA within groups, Control-1, p = 0.338; Control-2 p = 0.724). For the VPA grouped measurements, both demonstrated a steady and linear increase in t-PA release during the course of the ISP provocation (3–18 minutes, repeated measured ANOVA within groups, VPA-1, p = 0.003; VPA-2 p = 0.019).

**Fig 3:**
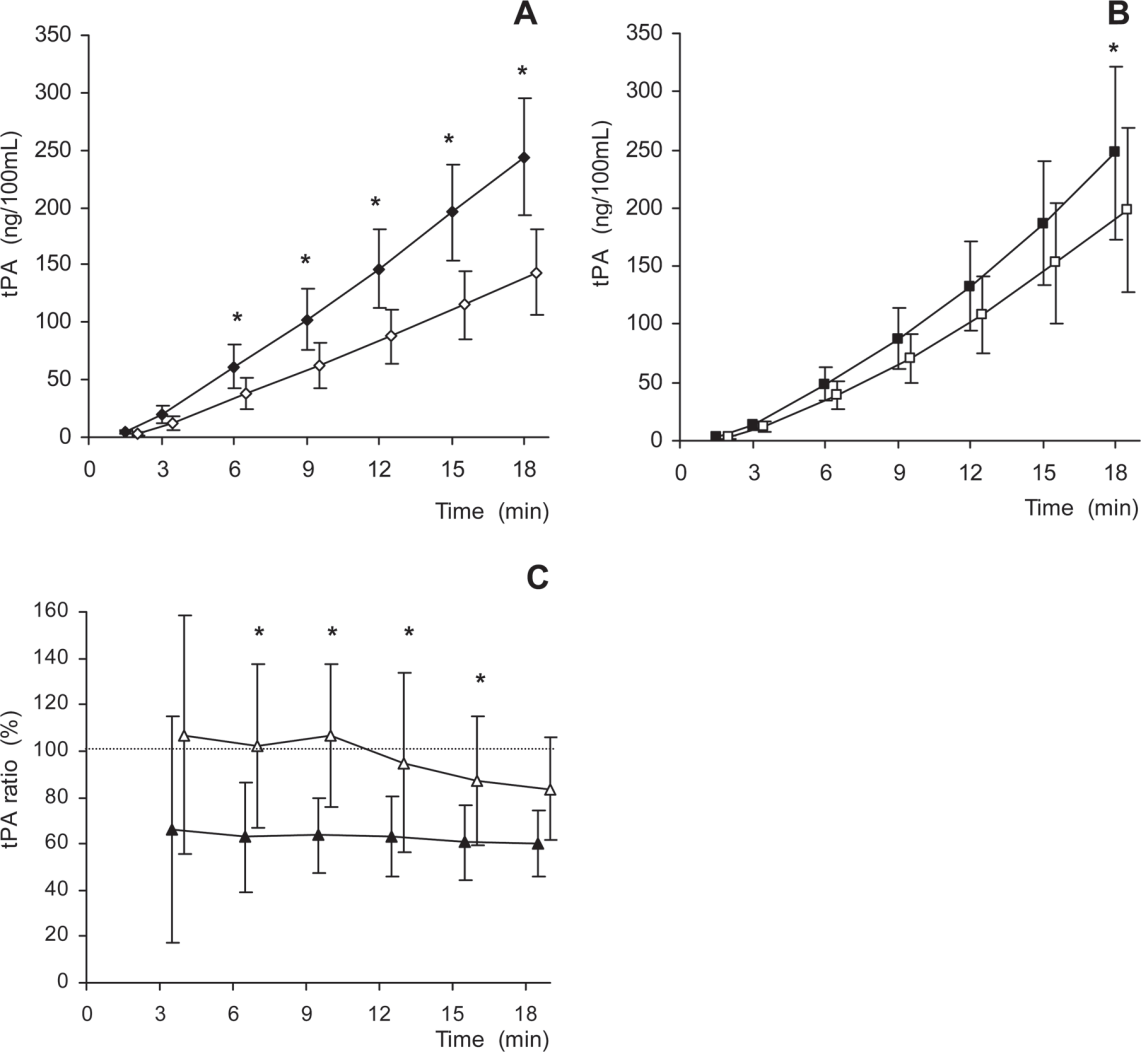
Exhaustion in cumulative t-PA release. In Panel A, the Control-1 (filled diamond, n = 16) and Control-2 (open diamond, n = 15) measurements show a pattern of exhaustion during the second stimulated t-PA release, starting early and continued throughout the sequence. Panel B shows the VPA treated group, with VPA-1 (filled square, n = 15) and VPA-2 (open square, n = 15) observations compared. There is no decrease observed in the second measurement until after 18 minutes. Panel C demonstrates a percent of the second measurement sequence compared to the first for both Control (filled triangles) and VPA (open triangles) (n = 13, where data was available for all measurements, treated and non-treated for the first and second sequences on both days). Minute 3 ratios reflect relatively small cumulative sums, possibly explaining the larger variation in the groups in Panel C. Data are presented as mean with 95% confidence intervals. * indicates p value less than 0.05 using repeated paired t tests.

A first finding is that paired comparisons at individual time points showed that there was a systematic difference and lower level for t-PA release from the first to the second grouped measurements for Control measurements. This same difference was not observed for the VPA treated grouped measurements until the end of 18 minutes. Comparisons to identify effects of repeated stimulation are presented in simplified analysis with cumulative t-PA release for grouped measurement sequences (Fig. 3). Concerning the first of the day ISP sequence, there was no observed effect of VPA on cumulative t-PA release (VPA-1 vs. Control-1, not shown together in a specific panel). On the other hand, concerning effects of VPA on t-PA release during repeated stimulation, exhaustion was clearly demonstrated in the Control grouped measurements, during the second measurement sequence, starting during early in the 20 minute sequence. For VPA measurements, signs of exhaustion were noted only late during the measurement sequence, first at 18 minutes. In order to directly compare the amount of exhaustion between the VPA and Control grouped measurements, each pair of values from individual subjects (Control-1 with Control 2, as well as VPA-1 with VPA-2) were used to form a grouped ratio of response. For the first period of the ISP stimulation, VPA demonstrated less exhaustion in contrast to the Control (differences in paired comparisons for minutes 3–15). Also notable is that there are no sign of change in exhaustion for Control during 20 minute sequence.

## Discussion

In this study we have shown that VPA influences endogenous fibrinolytic system expression in man, though there was no effect observed in baseline t-PA levels or during a first 20 minute ISP stimulation. Effects of VPA treatment were noted during repeated stimulation sequences, and are characterized as reduction in the amount of exhaustion. In combination with reduction in exhaustion during repeated and prolonged cumulative stimulated t-PA release, VPA caused a decrease in basal PAI-1 levels. This was an explorative study motivated by the idea that both of these effects in theory could have clinical relevance in the setting of recurring or prolonged thrombosis. While endothelial t-PA synthesis and storage was not measured directly with this study design, one can postulate that limitation of exhaustion in t-PA release caused by VPA could be a result of increased t-PA synthesis and availability, which is consistent with the presumed mechanism of HDACi effects on endothelial t-PA expression. These results confirm the hypothesis that VPA, with HDACi effects, can have potentially positive effects on expression of t-PA and PAI-1 as far as promoting more fibrinolytic effect. This in vivo confirmation of HDACi and VPA effects means that it is important to study further this potential future avenue of treatment.

Treatment with VPA did not affect all aspects of t-PA release. Resting baseline (constitutive) t-PA release was not affected by VPA, nor was the cumulative t-PA release during the initial stimulation affected by treatment, in line with findings reported in a healthy volunteer series [19]. Besides reducing exhaustion during repeated stimulation, the VPA treatment was also associated with differences in release patterns. During ISP stimulation, there was a progressively increasing pattern of t-PA release in the VPA-treated measurements over the course of the 20 minute stimulation that was not observed in the Control groups. The mechanism of this VPA-associated effect is not clear. These findings must be considered preliminary and hypothesis generating. The hypothesized mechanism of HDACi effects concerning t-PA expression was primarily concerned with regulation of gene transcription and t-PA synthesis. There are complex and intricate processes for storage, mobilization and release of t-PA, and if and how the VPA intervention has affected any of these processes in vivo is not known.

While VPA treatment has been demonstrated to alter t-PA gene expression through increase in gene transcription and synthesis of t-PA, leading to increased constitutive releases of t-PA from cultured human endothelial cells through a HDACi effect [15,16], this is the first report of an effect of HDACi on t-PA release capacity in man with atherosclerotic disease. We have previously shown in an *in vivo* porcine model, that t-PA release across the coronary vascular bed increased in response to a HDACi treatment. In that study, we did not observe any effect on baseline t-PA or PAI-1 values by VPA [18]. These findings support the idea that HDACi and VPA treatment can lead to increase in cellular synthesis, along with increased storage of t-PA in intracellular vesicles in endothelial cells. This can be associated with different patterns of resting and stimulated t-PA release.

Release patterns for t-PA may be regional and organ specific [24]. It may be expected that a forearm adrenergic-stimulated t-PA release pattern could differ from other models. This study was not designed to elucidate the mechanism of t-PA release, where endothelial cells in vivo exist in an environment of complex signaling and stimulation. Endothelial cells, beside their constitutive release of t-PA, upon stimulation, release t-PA that is available in stored intracellular vesicles that are prepared for release, though this by no means reflects the total amount of t-PA in different cell depots. Measuring t-PA release at rest or from a single stimulation may not reflect total t-PA synthesis. Prolonged or repeated stimulation of the endothelium is clinically relevant for patients with thrombotic tendencies, though single time-point stimulation also has relevance, since it is recognized that early and immediate local t-PA release has the most effect in preventing local thrombus formation [8,9]. The phenomenon of reduced t-PA release in response to repeated stimulation (exhaustion) has been described in a porcine model previously [25,26], but has not been clearly shown in clinical subjects to date.

The effect of VPA on global degree of histone acetylation has been well demonstrated in clinical material [27]. In this study design, we have employed a clinically established VPA dosing which is not expected to be toxic in another context. Plasma concentrations of VPA confirmed that the subjects received the intended treatment. Given the global aspect of HDACi effects, it may be expected that expression of proteins other than t-PA might occur with VPA treatment. The VPA treatment in our current findings led to significantly reduced PAI-1 expression. This effect of VPA has been noted previously on in vitro expression of PAI-1, and recently in a human model by others in our group [19,28]. This may be clinically relevant since fibrinolytic status in man is determined by t-PA/PAI-1 ratios. Reduced PAI-1 levels with sustained t-PA basal levels most certainly favors the endogenous fibirinolytic capacity. Treatment with VPA was associated with measureable but small reductions in fibrinogen and C-reactive protein levels, confirming a possible mild anti-inflammatory effect of VPA in this cohort, which has already been well demonstrated [29].

Concerning study design, this was not a randomized clinical study comparing two subject groups, but rather a clinical series with one subject group and a treatment cross-over protocol. This allowed subjects to serve as their own non-treated control for comparisons. Isoprenaline as stimulus does not provide a maximal stimulus of t-PA release [1] which potentially limits the value of comparison of the first and second t-PA stimulus sequence. Bradykinin and substance P are recognized to be more potent stimulators of t-PA release [30–32], and these were employed in our earlier work in this study series [19]. Substance P was our methodological first choice based on reproducibility of results in our hands, though it was not available for purchase commercially at the start of data collection. Collecting the Control measurement four weeks after treatment is judged by us to be acceptable, since global histone acetylation and deacetylation is recognized to be a dynamic process that reverses spontaneously over hours or days, once the HDACi substance is no longer present [20]. Only male subjects were included in order to limit the effect of endocrinological gender differences on t-PA release.

The clinical implications of potential improvement of t-PA reserve and release capacity are potentially profound, if this effect can be produced generally for patients with inadequate endogenous t-PA production and release. There is a clinical need to enhance the rapid and local fibrinolytic response in patients who have thrombotic tendencies, and this perhaps can be brought about through globally modifying gene transcription for t-PA. An epigenetic approach is appropriate for this type of treatment need.

### Conclusions

In summary, this explorative clinical study in post-myocardial infarction subjects tested HDACi and specifically VPA´s effect on one aspect of the fibrinolytic system: stimulated t-PA release. A mixed response emerged where static PAI-1 levels were suppressed. The first cumulative stimulated t-PA release did not increase with VPA treatment. However, VPA and HDACi intervention diminished and delayed exhaustion in stimulated t-PA release capacity which is observed in Control grouped measurements. We conclude that HCADi intervention shows promise as a possible intervention to improve endogenous fibrinolytic capacity, in patients with increased thromboembolic risk. More study is needed to establish clinical relevance of these findings as well as optimal dosing of a HDACi agent.

## Supporting Information

S1 Protocol Study protocol, English. (DOCX)

S2 Protocol Study protocol, native language (Swedish). (DOCX)

S1 Checklist TREND Checklist. (PDF)

S1 Dataset Numerical results corresponding to figures. (XLS)
