# Correction: Histone Deacetylase Inhibition Enhances Tissue Plasminogen Activator Release Capacity in Atherosclerotic Man

**DOI:** 10.1371/journal.pone.0128283

**Published:** 2015-05-08

**Authors:** Kristina Svennerholm, Michael Haney, Björn Biber, Erik Ulfhammer, Ott Saluveer, Pia Larsson, Elmir Omerovic, Sverker Jern, Niklas Bergh

In Fig 3C, the error bars are incorrect. Please see the corrected Fig 3 below.

**Fig 3:**
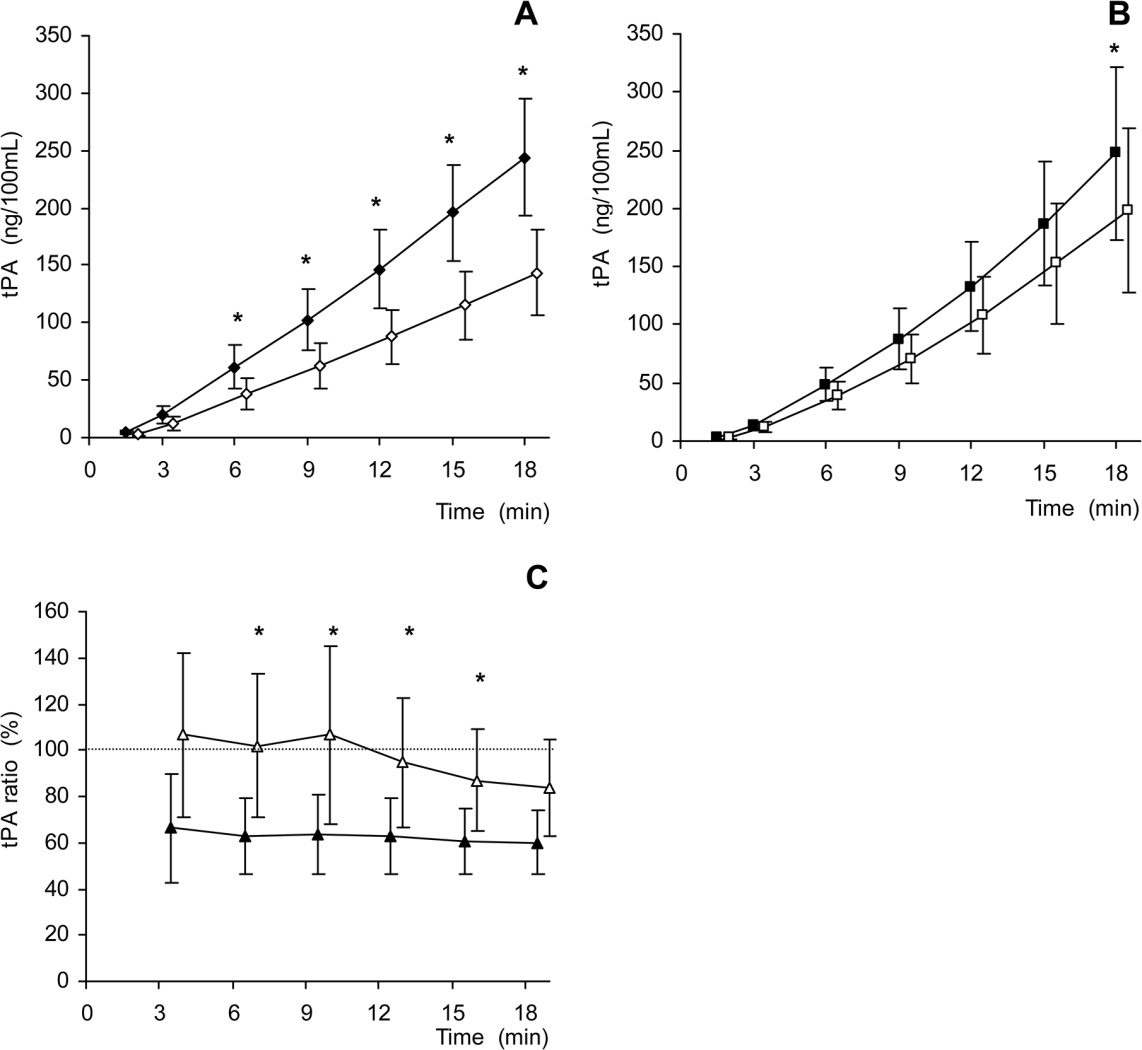
Exhaustion in cumulative t-PA release. In Panel A, the Control-1 (filled diamond, n = 16) and Control-2 (open diamond, n = 15) measurements show a pattern of exhaustion during the second stimulated t-PA release, starting early and continued throughout the sequence. Panel B shows the VPA treated group, with VPA-1 (filled square, n = 15) and VPA-2 (open square, n = 15) observations compared. There is no decrease observed in the second measurement until after 18 minutes. Panel C demonstrates a percent of the second measurement sequence compared to the first for both Control (filled triangles) and VPA (open triangles) (n = 13, where data was available for all measurements, treated and non-treated for the first and second sequences on both days). Minute 3 ratios reflect relatively small cumulative sums, possibly explaining the larger variation in the groups in Panel C. Data are presented as mean with 95% confidence intervals. * indicates p value less than 0.05 using repeated paired t tests.
