# Supplementary material for: Histone Deacetylase Inhibition Enhances Tissue Plasminogen Activator Release Capacity in Atherosclerotic Man
Source: PLoS One. 2015 Mar 25;10(3):e0121196. doi: 10.1371/journal.pone.0121196 (PMC4373842; doi:10.1371/journal.pone.0121196)
Supplement: S1 Protocol — (DOCX) [file pone.0121196.s001.docx]

|  | | Clinical study protocol | | |
| --- | --- | --- | --- | --- |
|  | | Substance: | | Valproic acid |
|  | | Study code: | | VPA-02 |
|  | | EUdraCT number | | 2012-004950-27 |
|  | | Date: | | 2012-11-15 |
|  | |  | |  |
|  | | | | |
| Effect of valproic acid on the fibrinolysis in humans with atherosclerotic disease | | | | |
|  |  | |  | |
| **Principal investigator:** | Dr Niklas Bergh Institution of Medicine  Department of molecular and clinical medicine The Wallenberg Laboratory, Sahlgrenska Univerisity Hospital, Bruna Stråket 16 413 45 Göteborg | | Phone 031 342 7574  Phone: 073 939 7415  Niklas.berg@gu.se | |
| **Location:** | The Wallenberg Laboratory  Bruna Stråket 16  Sahlgrenska Univerisity Hospital/SU  413 45 Göteborg | |  | |
| **Co-workers:** | Sverker Jern  Björn Biber  Björn Dahlöf  Pia Larsson  Ott Saluveer  Kristina Svennerholm | |  | |
| **Sponsor:** | Sahlgrenska University Hospital/SUS  413 45 Göteborg | |  | |

| **Amendment Nb.** |  | **Date:** |  | **Administrative change Nb.** |  | **Date:** |
| --- | --- | --- | --- | --- | --- | --- |
|  |  |  |  |  |  |  |
|  |  |  |  |  |  |  |

**Contact Information**

| **Principal investigator** | Dr. Niklas Bergh, MD. PhD The Wallenberg Laboratory, Sahlgrenska University Hospital, Bruna Stråket 16 413 45 Göteborg | phone 031 342 7574  phone: 073 939 7415  Niklas.berg@gu.se |
| --- | --- | --- |
| **Co-worker** | Prof. Björn Biber  Wallenberg Laboratory, Sahlgrenska Univerisity Hospital, Bruna Stråket 16 413 45 Göteborg | phone: 031-342 1000  Björn.biber@aniv.gu.se |
| **Co-worker** | Docent Björn Dahlöf  Wallenberg Laboratory, Sahlgrenska Univerisity Hospital, Bruna Stråket 16 413 45 Göteborg | phone: 031-342 1000  Bjorn.dahlof@a-plusscience.com |
| **Co-worker** | Dr. Pia Larsson  Wallenberg Laboratory, Sahlgrenska Univerisity Hospital, Bruna Stråket 16 413 45 Göteborg | phone: 031-342 1000  Pia.larsson@gu.se |
| **Co-worker** | Dr. Kristina Svennerholm  Wallenberg Laboratory, Sahlgrenska Univerisity Hospital, Bruna Stråket 16 413 45 Göteborg | phone: 031-342 1000  Kristina.svennerholm@vgregion.se |

| TABLE OF CONTENTS | Page |
| --- | --- |

To update TOC please use the GEL Toolbar AZ TOCs and Update all lists and delete hidden Bookmarks

TABLE OF CONTENTS 3

1. SYNOPSIS 6

ABBREVIATIONS AND DEFINITIONS 8

2. introduction 8

2.1 Background and rationalists 8

2.1.1 Rational for selection of dose of Isoprenaline 9

2.2 Research hypothesis 11

2.3 The benefit-risk assessment 11

2.4 Ethics 12

3. STUDY OBJECTIVES AND OUTPUT VARIABLES 12

3.1 Efficacy variables 12

3.1.1 Main variables 12

4. Study Design 12

4.1.1 Restrictions 14

4.1.2 Perfused forearm model 14

4.1.2.1 Study day I: 14

4.1.2.2 Study day II: 15

4.1.3 Stop criteria for continuing treatment 15

4.2 Follow up 15

5. Study population 16

5.1 Selection criteria 16

5.1.1 Rational for the chosen study design 16

5.2 Inclusion criteria 16

5.3 Exclusion criteria 17

5.4 Patient number, randomization, coding 17

5.5 Voluntarism and the criteria to end the study in advance 18

6. Treatment 18

6.1 Ergenyl Retard® 18

6.2 Isoprenalin 18

6.3 Dosing 19

6.3.1 Dilution scheme for Isoprenaline (0,2µg/ml) 19

6.4 Marking, preparation and storage 19

6.5 Disclaimer 19

6.6 Co-administration of other drugs 20

6.7 Adherence 20

7. measurement variables 20

7.1 Screening and data 20

7.2 Sampling and analysis of measured variables 20

7.2.1 Study Specific blood tests 21

7.2.2 Invasive examination of tPA release, in vivo measurement of blood flow 21

7.2.3 Blood at the screening visit 22

7.3 SAFETY 22

7.3.1 Adverse events 22

7.3.2 Serious adverse events 23

7.3.3 Suspected Unexpected Serious Adverse Reactions 23

7.3.4 Summary 24

8. Statistics 24

8.1 Power calculation 24

8.2 Analytical methods 24

9. SAMPLE AND DATA MANAGEMENT STUDY 24

9.1 Quality Control and Assurance 24

9.2 Data Management 25

9.2.1 Analysis of data 25

9.3 Biological material 25

9.4 Access to relevant securitystaff 26

9.5 Insurance 26

9.6 Timetable 26

9.6.1 Closed trial 26

9.7 Reports and Publications 26

10. REFERENCE LIST 27

APPENDIx

Appendix A Signature

Appendix B Definitions and further information on Safety

Appendix C Schedule for survey

# SYNOPSIS

**EudraCT number:** 2012-004950-27

**Study code:** VPA-02

**Principal investigator:** MD Niklas Bergh Department of molecular and clinical medicine The Wallenberg Laboratory, Sahlgrenska University Hospital, Bruna Stråket 16 413 45 Göteborg

**Timeplan: Q1 2013 to Q4 2014**

**Title: Effect of valproic acid (VPA) on fibrinolysis in humans with atherosclerosis**

**Objective:** To investigate whether upregulation of tPA production by valproic acid results in increased tPA release from the endothelium in humans.

**Study design and study drug:** This is a single-centre, open (blinded for data analysis), randomized, crossover study of 30 men and women with severe coronary artery disease who underwent an emergency PCI for at least a year ago. The subjects will be evaluated for the ability of endothelial cells to release tPA, before and after treatment with VPA in addition to their regular medications. An invasive test, the so-called perfused forearm model will be used.

**Inclusion criteria**

1. Informed consent from patient
2. Men and women ages 50 –85 years
3. Treated for acute myocardial infarction for ≥ 1 year ago
4. Severe atherosclerotic disease (3-vessel disease, syntax scores above 20)
5. Non-smokers
6. No other anticoagulant therapy except aspirin

**Exclusion criteria:**

1. Smoking
2. BMI (body mass index) > 35 kg/m²
3. Epilepsy,
4. Uncontrolled hypertension
5. Malignancy
6. Psychiatric disorder
7. Alcoholism
8. All forms of chronic illness with medications that are contraindicated to combine with Ergenyl Retard
9. Acute infection
10. Difficulties in implementing the study in view of the inability to lie still on a bed due to the general condition or obvious difficulties with putting vein / artery catheter
11. Interaction problems between Ergenyl and other patient medication
12. Known hypersensitivity to valproic acid or other constituent component
13. Suspected or actual inability to perceive the study information and/or instruction

**Efficacy Variables:** Blood flow, blood pressure, heart rate, fibrinolytic components, lactate levels

**Statistics:** It is estimated that 30 individuals should be enough to reveal a biologically relevant difference (roughly 50%) in this experimental test set-up. The subjects are their own controls, which significantly increases the power (probability of detecting a difference) since we avoid inter-individual variation. Standard methods will be used for statistical analyses of the study data.

ABBREVIATIONS AND DEFINITIONS

| AE | Adverse event (Appendix B) |
| --- | --- |
| CRF | Case Report Form |
| ECG | Electrocardiogram |
| EC | Ethics Committee, synonymous to Institutional Review Board (IRB) and Independent Ethics Committee (IEC) |
| GCP | Good Clinical Practice |
| ICH | International Conference on Harmonisation |
| OAE | Other Significant Adverse Event (ie, adverse events of particular clinical importance, other than SAEs and those AEs leading to discontinuation of the subject from study treatment; see definition in Appendix B |
| SAE | Serious adverse event (Appendix B) |
| VPA | Valproic acid |

# Introduction

## Background and Rational

Tissue plasminogen activator (tPA) is a protein that starts a blood clot dissolving mechanisms and are thus both involved in processes that prevent the onset of and dissolve blood clots in blood vessels. TPA activity is regulated by inhibitors, the most important is PAI-1. The cells that form the innermost layer of the blood vessel wall (endothelial cells) produce tPA and continuously releases a small amount of the protein into the bloodstream. Since tPA is stored in the endothelial cells, stimulation of the endothelium may result in t-PA release. Upon stimulation t-PA concentration may locally increase 20-fold in close proximity to the blood clot.

Our research group has since 1994 had an experimental set-up, "the perfused forearm model", where the ability to release tPA can be studied in humans. For example, we found that patients with hypertension and renal patients (these patients have an increased risk of cardiovascular disease), have severely impaired ability to release tPA upon stimulation.

Furthermore, our studies have shown that treatment of hypertension improves the fibrinolytic system but the underlying mechanisms are incompletely mapped. The problem has been that there has not been a tool to stimulate tPA synthesis.

Our research group has recently found that valproic acid (VPA), which is a well-established drug for the treatment of patients with epilepsy, dramatically upregulates tPA gene in cultured endothelial cells, resulting in both increased mRNA and protein level. This upregulation occurs within one day and is seen in both human endothelial cells from coronary artery, umbilical vein and aorta. Upregulation of tPA is around 10-15 times above the basal level for both mRNA and protein. The concentration of VPA used in the culture medium is at the same level as used in patients with epilepsy, i.e. therapeutic levels. It is very important to determine whether the upregulation of tPA also applies to man, for in that case we would possibly have a potent drug to stimulate the endogenous fibrinolysis.

We have recently conducted a study in healthy volunteers, where we investigated the acute ability for t-PA release before and after VPA treatment. This study showed no severe adverse events with either treatment or the experimental setup. In this study, we saw no significant up-regulation of t-PA. However, we saw a significant inhibition of PAI-1 levels. That we failed to see any significant effect on t-PA regulation may be due to that these normal subjects had an intact fibrinolytic system and hence it is difficult to create a supra-normal fibrinolytic defense compared to normalizing a defective system. What strengthens our hypothesis is that VPA had no effect on PAI-1 levels. Usually, t-PA and PAI-1 is regulated by the same stress, but in opposite directions.

In the present study, we have chosen to include patients with manifest atherosclerotic disease and prolong treatment time to 4 weeks to detect a difference between groups. Cardiovascular disease patients often have a low-grade inflammatory activity resulting in increased cytokine levels. We have shown in in vitro experiments that cytokines counteracts and delays the effect of VPA on tPA regulation. It is reasonable to expect similar relationships in humans

The main study variables in the study is the release of tPA, plasma levels of PAI-1 as well as other measures of vascular function such as blood flow changes during stimulation. Intravascular administration of isoprenaline will be used for vaso-dilation stimulation. Isoprenaline has been utilized in a number of experimental situations to produce dose-dependent β-adrenergic activation.

The study is expected to increase our knowledge on the regulation of the body's defense mechanisms against blood clot formation by newly discovered regulatory mechanisms

.

### Rationale for dose selection

Ergenyl Retard® depot tablets is an antiepileptic drug for the treatment of generalized seizures. We have chosen a dose of 2x500 mg ie in the lower part of the recommended dose range for epilepsy therapy to minimize undesirable side effects. In our previous study in healthy volunteers treated with the same dose, there was no patient who experienced any side-effects or withdrew prematurely. We judge that this dose, based on in vitro data, should have the effect on tPA expression.

Isoprenaline (isoproterenol) is a direct-acting non-selective drugs that act as an agonist of adrenergic membrane receptors type of β1 and β2. Its affinity for the α-adrenergic receptors is very low. Upon intravenous administration of isoprenaline there is a dose-dependent reduction of muscle tone in vascular smooth muscle, with consequent dilatation in particular vascular beds in skeletal muscle, kidney and intestine. Reduction in diastolic blood pressure may be noted with increasing dose, whereas systolic blood pressure is either unchanged or increased. The latter reflects a concurrent increase in cardiac output due isoprenalins positive inotropic and chronotropic effects. A dose-dependent increase in heart rate is thus a clear element in isoprenalins effect profile. Isoprenaline reduces the tonus of the smooth muscle bronchial tree and gastro-intestinal tract. Isoprenaline is rapidly metabolised by COMT (catechol-O-methyltransferase), mainly in the liver.

Clinical use of isoprenaline: Isoprenaline has been used since the early 1950s (Nathanson & Miller 1952; Reed Wood 1969) as intravenous drug for increase in heart rate when bradycardia or AV block, especially in anticipation of implantation of pacemakers and patients with torsade de pointes. For this indication, isoprenaline is still used in emergency medicine, cardiology, intensive care and anesthesiology. For the treatment of asthma (Hume and Gandevia 1957) and circulatory failure (MacLean et al. 1,965; Wosornu and Easmon 1970) isoprenaline has gradually been replaced by other drugs that have a more receptor-specific adrenergic agonists. (Crompton 2006; Santman 1992). Thus, consumption of isoprenaline has decreased overall, and the substance is now available in Sweden only as a licensed medicine. What is clear is that there is a very comprehensive and unambiguous clinical documentation on the clinical usefulness of isoprenaline and its side effects. The latter is confined to such general dose-dependent acute reactions consistent with what is normally seen after endogenous β-adrenergic activation (ie, tachycardia, vessel dilation and increased contractility of the myocardium).

Examples of experimental use of isoprenaline in homo: Isoprenaline has been used in several experimental situations to produce a dose-dependent β-adrenergic activation, such as mapping for other cardiovascular-active drugs (Woltz et al., 1995), studies of the adrenergic control of temperature control (Stob et al., 2007), tissue lipolysis (Jock et al., 2007), inhibition of NO synthase (Schmetterer et al., 1999) and interoceptive awareness (Khalsa et al. 2009) (table 1)

.

Our planned use of isoprenaline in this study: We intends to use short intravascular administration of isoprenaline, where the planned dose range is very well consistent with (or lower than) the doses reported in the accumulated literature database and no adverse or unexpected side effects have been documented (Shindler et al.2004, Stein et al. 1998 Giannarelli et al. 2008, DeSouza et al., 2002, Chandler et al 1995, Cardillo et al 199), Table 1. A possible, but unexpected, rise in heart rate and / or average blood pressure can, in our own extensive clinical experience and the available literature database, rapidly be controlled by dose reduction. The efficacy of isoprenaline is, by its rapid degradation, a matter of a few minutes.

| ***Table 1 The rational of dose selection for Isoprenaline*** | | | | | | |
| --- | --- | --- | --- | --- | --- | --- |
|  | **Schindler^1^** | **Stein^2^** | **Giannarelli^3^** | **DeSouza^4^** | **Chandler^5^** | **Cardillo^6^** |
| ***Dose Isopren*** | 2-514 ng/min | 10 - 400 ng/min | 0,03  µg/100ml/min  (= 30 ng) | 5-10-20  ng/100ml/min | 7 och 35 ng/kg/min | 50, 100, 200  ng/min |
| ***Infusion time (min)*** | 10 | 7 | 15 | 5 | 18 min every dose | 5 |
| ***t-PA release*** | Not measured | + 25  ng/100ml/min  (0,7 → 26,2 ) | + 2  ng/100ml/min | Not measured | 77±35 -> 106±49 pmol/L | Not measured |
| ***Q forearm*** |  | 1,4 → 9,7  ml/100ml/min | 5→15  ml/100ml/min | 4→12→14→17  ml/100ml/min | 3 ±1 -> 6±1 ml/s | 2→6→9→11  ml/100ml/min |
| ***HR*** | 60 →70 | 60 →64 | not reported | not reported | increased 73%  59 - >102 | MAP unchanced |
| ***Vein size*** | + 40 – 70 % | not reported | not reported | not reported | not reported | not reported |
| ***Method*** | Vein diameter | plethysmography | plethysmography | plethysmography | plethysmography | plethysmography |

**1.** Schindler C, Dobrev D, Grossmann M, Francke K, Pittrow D, Kirch W. Mechanisms of beta-adrenergic receptor-mediated venodilation in humans. *Clin Pharmacol Ther* 2004;75:49-59.

**2.** Stein CM, Brown N, Vaughan DE, Lang CC, Wood AJ. Regulation of local tissue-type plasminogen activator release by endothelium-dependent and endothelium-independent agonists in human vasculature. *J Am Coll Cardiol* 1998;32:117-122.

**3.** Giannarelli C, Virdis A, De Negri F, Duranti E, Magagna A, Ghiadoni L, Salvetti A, Taddei S. Tissue-type plasminogen activator release in healthy subjects and hypertensive patients: relationship with beta-adrenergic receptors and the nitric oxide pathway. *Hypertension* 2008;52:314-321.

**4.** DeSouza CA, Clevenger CM, Greiner JJ, Smith DT, Hoetzer GL, Shapiro LF, Stauffer BL. Evidence for agonist-specific endothelial vasodilator dysfunction with ageing in healthy humans. *J Physiol* 2002;542:255-262.

**5.** Chandler WL, Levy WC, Stratton JR. The circulatory regulation of TPA and UPA secretion, clearance, and inhibition during exercise and during the infusion of isoproterenol and phenylephrine. *Circulation* 1995;92:2984-2994.

6. Cardillo C, Kilcoyne C, Quyyumi A, Cannon R& Panza J. Decreased vasodilator response to isoproterenol during nitric oxide inhibition in man. Hypertension 1997; 30: 918-921.

## Research Hypothesis

Can upregulation of tPA production by valproic acid result in increased tPA release from the endothelium in humans?

If our hypothesis proves true, this could be a new way of preventing heart attack, ischemic stroke, deep vein thrombosis and pulmonary embolism.

## Benefit-risk assessment

The risks of participation consists of possible complications associated with catheterisation of the artery. There may be a risk of potentially serious complication such as occlusion of the vessel wall. It is possible to diagnose and fix this adverse event if it occurs. Another possible serious complication would be if the brachial nerve is seriously damaged by the needle. At anticipated nerve damage a neurologist will be engaged for specialist assessment. We have never experienced any of the above complications and therefore considers these theoretical risks very small.

The more probable complication that can occur is bleeding after the artery catheter is removed. The bleeding gives a bruise that may initially be painful. However, these symptoms are always transient as the hematoma is resorbed over time. We have established procedures to minimize this complication which includes extended compression time. We document each catheterization.

In our previous study in healthy volunteers treated with the same dose (2x500 mg / day), there was no patient who experienced any sideeffects or withdrew prematurely. We will closely monitor our patients during the treatment and possible side effects, carefully consider whether the study drug should be stopped or not.

The subject will receive both verbal and written information about how they should proceed if it starts to bleed, if it get swollen, start to ache or if side effects occur. We will give 24-hour emergency phone number to call if complications arise.

Infusion of Isoprenaline gives no general effect. Treatment with valproate has a range of side effects described / risks during long-term use, but only when the patient is treated for a month, we believe that the risks of serious complications is small.

The subjects really have no personal benefit from participation in the study, more than that they have a medical examination, proper care and control of routine blood tests.

We have a secure method of investigation which is the only method that provides accurate information about the body's own defenses against the formation of blood clots. We have previously evaluated a non-invasive test (ishemitest) as a method to stimulate tPA release but found that this is arbitrary. Valproic acid, which is a proven and safe drug in the treatment of epilepsy, will be given as a pill for a month with the approved dosage. In such a short period of treatment, we expect no serious complications, such as bleeding. Given that new regulatory mechanisms for tPA release and production will be highlighted and that there are no available animal models, we believe that the study involves a small risk compared with the important issues that it will provide answers to.

## Ethics

The study will be conducted in accordance with ICH Good Clinical Practice (GCP). International and local regulatory requirements that are based on the Helsinki Declaration on ethical standards for research involving human subjects will be followed.

Written consent and / or a positive review must be obtained from the Regional Ethical Review Board prior to study entry.

The investigator must assure that the study patients were given written and verbal information about the study design, purpose, possible risks and potential benefits. A copy of a signed agreement from the patient is kept by the principle investigator.

# STUDY PURPOSE AND EFFECT CHARACTERISTICS

The primary purpose of this study is to examine whether upregulation of tPA production by valproic acid results in increased tPA release from the endothelium in humans.

Primary endpoint: tPA release

## Effect variables

### Main variables

Blood flow, blood pressure, heart rate, factors associated with the fibrinolytic system and lactate levels will be analyzed.

# Study Design

This is a single-center, open (blinded for data analysis), randomized, crossover study in 30 patients with severe coronary artery disease who underwent an emergency PCI for at least a year ago. The subjects will be evaluated for the ability of endothelial cells to release tPA before and after treatment with VPA in addition to their regular medications. An invasive test, the so-called perfused forearm model will be used.

According routine at the clinic, patients have double antiplatelet therapy with ticagrelor (Brilique) and Aspirin (Trombyl) associated with acute PCI. Ticagrelor is normally discontinued after 12 months of treatment while Aspirin persist indefinitely. Because we do not want the patients to be on dual antiplatelet therapy while the forearm study is performed, due to the increased risk of bleeding, it must therefore have been at least 12 months after PCI treatment.

Visit 1 At the first visit, the subjects are given written and oral information of the study managers and after signed informed consent is given, they are included in the study. Patients undergo a thorough examination of blood pressure measurement, ECG, clinical examination and basic blood tests.

Subjects were randomized thereafter to either of two treatment groups. One group started 4 weeks VPA treatment while the other group did not receive any treatment at all. The date for study day one I set. The second group starts with VPA treatment 4 weeks prior to study day II while the first group have no further study treatment There is two weeks washout period placed in the middle of the study (Figure 1).

***Figure 1 Study design***

VPA n=15

VPA n=15

wash-out

No treatment n=15

No treatment n=15

R

Week: 0 1 2 3 4 5 6 7 8 9 10

| ***Table 2 study activities*** | | | | |
| --- | --- | --- | --- | --- |
|  | Visit 1 | Visit 2 | Visit 3 | Visit 4 |
| week: | 0 | 4 | 6 | 10 |
| Informed consent | X |  |  |  |
| Age, sex | X |  |  |  |
| Medical history | X |  |  |  |
| Smoking/alcohol | X |  |  |  |
| Weight/length | X |  |  |  |
| Pulse/blood pressure | X | X |  | X |
| ECG | X | X |  | X |
| Medical exam | X |  |  |  |
| Blood test (table 2) | X | X* |  |  |
| VPA | X |  | X |  |
| Perfused forearm model |  | X |  | X |
| plethysmography |  | X |  | X |
| Serum concentration of valproic acid |  | X |  | X |
| Study specific blood samples (7.2.1) |  | X |  | X |
| Serious reactions and events | X |  |  |  |

* fasted (cholesterol, LDL, HDL, TG)

Visit 2 and 4. The release of tPA is measured in the non-dominant forearm. Release is stimulated by intra-arterial infusion of Isoprenaline 200 and 400 ng / min for 20 minutes. For those who have received 4 weeks of oral treatment with VPA, the study mediation is now discontinued. After about 2 weeks of washout without medication change the subject change treatment group (VPA or no treatment in the following 4 weeks). Thereafter, there is a new perfused forearm attempts. Time between Study 1 and 2 is approximately 6 weeks.

The patient will come to the study department at 8 am on the morning of study and must have been fasting since midnight. Regular use and study medication (VPA) may be taken with a glass of water no later than 7.00 am the moring of the study examination. See restrictions 4.1.1. Blood concentrations of valproic acid is monitored in all subjects each study day. Other blood samples are taken at the beginning of Study I: fB-glucose, cholesterol, LDL, HDL, triglycerides.

Visit 3. On this visit, VPA medicine is shared to the treatment group 2.

### Restrictions

- The patients should be fasting from midnight before the study day
- No intake of tea or coffee in the morning on study days
- Regular morning medications may be taken with a glass of water
- No anti-inflammatory drugs (such as Voltaren, Pronaxen, Nurofen, Confortid, Brufen) days prior to the study or on the survey day.
- Pain management if required is only paracetamol
- Caffeine or theophylline-containing diet (bananas, coffee, tea, chocolate) is not permitted for 12 hours before study day
- No alcohol within 24 hours before a study day
- No vitamin last 10 days
- No strenuous physical exercise 24 hours before the study day

### Perfused forearm model

#### Study day I:

The subject is expected to lie down to rest and relax as best they can during the investigation, but may listen to music or the radio if desired. Venflons are introduced in both arms deep antecubital veins. The arterial catheter is placed in the non-dominant arm in artery brachialis. The plethysmograf is connected to both arms for measuring the local blood flow. Blood concentrations of valproate are monitored in all subjects.

When all equipment is connected, the test subject rest for 30 minutes before the actual investigation begins. It goes as follows: blood samples are taken simultaneously from the arterial and venous catheters and rest blodd flow is recorded to evaluate the basal release level of tPA and blood flow. Next follows an intra-arterial infusion of Isoprenaline 200 ng / min, 1 ml / min. Blood flow measurements and blood samples are collected repeatedly during the 20-minute infusion to evaluate tPA release and blood flow increase in the forearm. Then the intra-arterial infusuion of Isoprenaline 400 ng / min, 1 ml / min is started. Blood flow measurements and blood samples are collected repeatedly during the 20-minute infusion to evaluate tPA release and blood flow increase in the forearm. During the following 10 minutes after completion of the infusion, again basic tests to monitor tPA basal levels are collected. After completing the examination all equipment is removed. The venous needles are first removed. Finally the artery catheter is pulled out and insertion site is compressed for 20 minutes by the responsible physician.

Study medicine for Group 1 is discontinued.

Total time of the survey is about 3 hours including preparation. See Section 7.2 for sampling schedule. Ss schedule for invasive investigation in Appendix C..

#### Study day II:

6-7 weeks after study day I the patients will return for study day II. This is implemented in the same way as study I. Blood concentrations of valproate is monitored in all subjects. Study medicine for Group 2 is discontinued.

### Stopping Criteria for continued medication

- The occurrence of adverse effects of the study drug or study procedure.
- Unwanted effects unrelated to the study drug or study procedure.
- The subject's own desire to discontinue study participation.

If any of the side effects such as anaphylaxis or hypersensitivity following administration does occur it is dealt with in the routine care and follow-up in the usual way

## Follow up

The patient will be in the hospital just before surgery and one hour after completion of the investigation. Careful monitoring of possible side effects can be identified and addressed immediately. If patients have to seek emergency care for bleeding problems from the catheterisation of the artery they have a note regarding study design to show to the physician. Patients will receive contact details for responsible study physician about any non-acute side effects and / or questions for study participation. Study Responsible physicians will be reachable around the clock.

Patients will after investigation be treated in the usual manner in the medical and health care

# study population

## Selection criteria

We target individuals with known severe coronary disease (age 50-85) treated for an acute myocardial infarction at the Sahlgrenska University Hospital for more than a year ago. In connection with the previous hospitalization, persons are registered in Riks-HIA / SCAAR registry and through this registry, patients will be recruited.

Selection criteria from the register is that they must have been treated for acute myocardial infarction over a year ago, have an extended coronary atherosclerosis disease, and non-smokers. We estimate that we will produce approximately 400 individuals to whom we will send a letter of offer to participate in our study (see letter to the subject). We will need 30 patients in order to fulfill the purpose of the study.

If they are interested, contact us and we will call them for a first visit where additional oral and written information given. After signed consent may undergo a basic physical examination by the physician in charge and they are checked for inclusion and exclusion

### Rationale for the chosen study design

The reason that we choose to recruit patients through the National-HIA / SCAAR registry is that we want to have as homogeneous a population as possible in order to most easily be able to demonstrate an effect with as few individuals as possible

The reason it must have gone at least 12 months since the acute myocardial infarction is that patients initially receive double platelet inhibition for 12 months, most often the combination of ticagrelor / Clopidogrel (Brilique / Plavix) and aspirin (Trombyl). When a year has passed, they are treated with only Trombyl. We do not want patients to stand on dual antiplatelet therapy because it increases the risk of bleeding. Other standard treatment that these patients usually receive is beta-blockade, diuretics, ACE / Angiotensin converting enzyme inhibitors and statins, which are not deemed to be an obstacle for study participation

## Inclusion criteria

Following inclusion criteria must be met:

1. The patient has given informed consent
2. Men and women aged 50 -85 years
3. Treated for acute myocardial infarction for ≥ 1 year ago
4. Extensive coronary atherosclerosis (3-vessel disease, syntax score of 20)
5. Non-smoker
6. No other anticoagulant apart Aspirin
7. Women of childbearing potential should use effective contraception during treatment or use sexual abstinence

## Exclusion criteria

The subjects may not be included in the study if the following exclusion criteria are met:

# Smoking

# BMI (body mass index)> 35 kg / m²

# epilepsy,

# uncontrolled hypertension

# malignancy

# mental disorder

# alcoholism

# All forms of chronic illness with medications that are contraindicated to combine with Ergenyl

# acute infection

# Difficulties in implementing the study in view of the inability to lie still on a bed because of the general condition or obvious difficulties with putting vein / artery catheter

# Interaction Problems between Ergenyl and the patient's other medications

# Known hypersensitivity to valproic acid or other constituent component

# Pregnancy and breast-feeding.

# Suspected or actual inability to perceive the study of information and instruction

## Patient number, randomization, coding

The investigator must assure that the study patients were given written and verbal information about the study design, purpose, possible risks and potential benefits before any study-related investigation begins. The principle investigator keeps a copy of a signed agreement from the patient.

After the subject signed a consent to participate and met all inclusion and exclusion criteria, he/she are assigned a unique study number / code recorded in CRF and on all test tube labels, questionnaires and hospital records. Patients excluded for medical or technical reasons, may not be recruited.

The study is open with respect to the treatment of VPA but blinded for analysis of outcomes. The code list is kept locked up under the procedures of the institution and is not be broken until all analyzes are made. The code list may be available only to those persons who are directly involved in the research project. Un-identified data will be processed in principle investigators password protected computer.

## Voluntary and criteria to end the study in advance

Study patients may at any time, without cause and without stating specific reasons, to discontinue participating in the study. The study doctor may decide to discontinue study participation by medical or administrative reasons (eg, identified risk for the patient, improperly included patient, serious protocol deviations). If a patient discontinued participation, he/she should be asked for side effects. Even the manufacturer of the study drug may withdraw the drug product, in which case this can lead to the study in its entirety may be interrupted

# Treatment

Ergenyl Retard and isoprenaline will be used in the study (Table 2).

| ***Tabell 3 Study drug*** | | |
| --- | --- | --- |
| **Product** | **Dose and administration** | **Manufacture** |
| Ergenyl Retard® | 500 mg, 1 tablets twice daily for 4 weeks | Sanofi |
| Isoprenalin | Solution for infusion 0.2 mg / ml, infused over 20 minutes. extemporaneous preparation | Hospira AB/Pharmel AB/ Oriola/Kronans Droghandel |

## Ergenyl Retard®

Ergenyl Retard® depot tablets an antiepileptic drug for the treatment of generalized seizures. The most commonly reported adverse reactions associated with the intake of this drug are gastrointestinal side effects and tremor which affects between 1-10% during long-term treatment. Unusual side effects are bleeding and liver involvement in such a short time. The medication should not be used in people with impaired liver function, hypersensitivity to sodium valproate or any of the excipients or porphyria.

## Isoprenaline

Isoprenaline is a direct-acting non-selectively vasoactive drugs that act as an agonist of adrenergic membrane receptors type of β1 and β2. Isoprenaline is rapidly metabolized primarily in the liver. Increases heart rate, stroke volume and coronary perfusion. Reduces peripheral resistance and hence the diastolic blood pressure. It is mainly used in the clinic at AV block II or III and before pacing and even to heart and heart-lung transplant patients to stimulate the heart rate the first postoperative day. Isoprenaline ordered through the hospital pharmacy as licensed preparations.

## Dosage

Ergenyl Retard® release tablets will be given according to the manufacturer's intructions (see FASS) ie 1 tablet of 500 mg in the morning and evening for 4 weeks. The last dose is taken at the respective Study Day tomorrow. The doses (200 and 400 ng / min) of isoprenaline is adapted so that the effects will be limited to the forearm, ie there will be no effect on systemic level

### Dilution Scheme for isoprenaline (0,2μg / ml)

Isoprenaline delivered in a dose of 0.2 mg / ml

1 ml Isuprel 0,2 mg/ml (=200 ug/ml =200 000 ng/ml)

1 ml Isuprel  = 200 000 ng, spädes i 1000 ml NaCl = 200 ng/ml

2 ml Isuprel diluted in 1000 ml NaCl = 400 ng/ml

## Labelling, preparation and storage

Isoprenaline ordered through the hospital pharmacy as licensed preparations and stored in an outer packaging which will be marked with study code, randomizer, name of study medication, dosage, strength and quantity, instructions, name of the responsible physician, expiration date, storage instructions, "for clinical trial", "kept reach of children "and used only for study purposes. Isoprenaline will be prepared according to the manufacturer's instructions in direct connection to the trial.

Ergenyl Retard tablets are obtained from the hospital pharmacy to the ward in their original packaging. These are stored in a common outer packaging which will be marked in accordance with the above information and be used only for study purposes. Each patient will be assigned a jar = 50 tablets at visits 1 and 3, respectively Each bottle is labeled with the label with the above information

## Responsability

All study medicine is stored during the study at the Wallenberg Laboratory in a locked cabinet that only study managers have access to. The investigator is responsible for the study drugs used in the study and that they are used only as described in the study protocol. Unused study drug will be destroyed and not used for other purposes. At the end of the study, it must be possible to trace all study medications. Tablet count will occur at Visit 2 and 4 respectively

## Co-administration of other drugs

Patients will be able to maintain their regular medication during the study, such as beta blockers, diuretics, ACE / angiotensin II inhibitors and statins. Medications and treatments that are considered vital for the patient and does not affect the study results will be given at the same time as the study drug.

All medications at the start of the study and any additional medications or altered prescriptions will be recorded in the CRF

## Adherence

When infusions of isoprenaline will occur in hospital clinic, we expect that compliance is complete. Isoprenaline is normally given in the routine care and according to the manufacturer's instructions will be followed.

Tablet counting after 4 weeks will be made and compared against distribuated tablets.

# measurement variables

## Screening and personal data

Study participants submit information at the first visit of

• Age, sex, weight, height

• Smoking

• Current and previous diseases, cardiovascular genetics, possible hypersensitivity / allergy

• Ongoing medication

• Clinical examination including heart, lungs, abdomen

• Heart rate, blood pressure, ECG

• Basal blood sampling. Blood samples to be analyzed before the study: blood count, elctrolytes, liver status, PK, APTT, cholesterol, LDL, triglycerides, glucose, s valproate (see Table 3)

All data entered in each patient CRF

## Sampling and analysis of measured variables

In total, approximately 440 ml of blood will be taken at Visit 1 and during the two study days

### Study-specific blood samples

Study-specific blood samples (400 ml) will be taken during the two study days as shown in Figure 2 and analyzed for the effect of VPA on fibrinolysis. The samples will be analyzed at the Wallenberg Laboratory, Sahlgrenska University Hospital. Research-based samples that tPA and PAI-1 are taken directly in coded tubes and then stored in coded form blood flow, blood pressure, heart rate, factors of the fibrinolytic system and lactate levels will be analysed

***Figure 2 Sampling Scheme for isoprenaline infusion study days***

Base 1, 15 min

Isoprenalin 200/400nmol/min för 20 min

Base 2, 10 min

min

1,5

3

6

9

12

15

18

10

5

2

5

10

art

ven

3

4

5

6

7

8

9

2

1

10

11

12

Nr

Study-specific samples will be handled and stored in accordance with the Biobank Act (see 9.3). The samples will be processed in a blinded with only a code number as indicator.

### invasive examination of tPA release in vivo measurement of blood flow

Equipment for measuring blood flow, known as plethysmography, are connected to the subject. This consists of a thin plethysomograf thread wrapped around both forearms and blood pressure manchets are linked on the upper arms. To register the flow of blood they are pumped briefly up repeatedly. The patient is also connected to ECG and blood pressure monitors (through the arterial catheter) allowing continuous recording of these parameters. The current tPA release calculated retrospectively by multiplying the instantaneous concentration difference between artery and vein with the current forearm plasma flow (which in turn is calculated from the forearm blood flow).

The blood flow during rest is registered first to evaluate the basal release level of tPA. At baseline, blood samples were drawn simultaneously from the vein and artery. The first 2 ml discarded (slush), then preferred 2 mL of chilled syringe, which is emptied in Stabilyte tube, mixed 4-5 times and then placed on ice until centrifugation. Then start an intra-arterial infusion of Isoprenaline 200 ng / min. Blood flow measurements and venous blood samples were collected repeatedly during the 20-minute infusion to evaluate tPA release and blood flow increase in the forearm. Then start an intra-arterial infusion of Isoprenaline 400 ng / min. Blood flow measurements and venous blood samples were collected repeatedly during the 20-minute infusion to evaluate tPA release and blood flow increase in the forearm. During the following 10 minutes of completed infusions taken again basic tests to monitor tPA release in the process. The subject is expected to rest and relax as best they can during the investigation, but may listen to music or the radio if desired. Drawn amount of blood during the experiment is 100 ml.

### Blood at the screening visit

Blood samples (40 ml) for screening is taken at Visit 1 (Table 3). These are sent to the clinical chemical laboratory at Sahlgrenska University Hospital for analysis. Laboratory samples were recorded in the usual FLEX lab

| ***Table 4 Blood-screen*** |  |
| --- | --- |
| ***Clinical Chemistry*** | ***Haematology*** |
| S-Sodium (Na) | B-hemoglobin (Hb) |
| S-Kaliun (K) | B-leukocytes (WBC) |
| S-Creatinine | B-Platelets (TPK) |
| S-hsCRP |  |
| S-glucose |  |
| S-Alkaline phosphatase (ALP) |  |
| S-Alanine aminotransferase (ALT) |  |
| S-Aspartan aminotransferase AST) |  |
| S-PK |  |
| S-APTT |  |
| P-total cholesterol |  |
| S-valproate |  |
| P-TG, HDL, LDL |  |

## Safety

Responsible physicians must ensure that procedures and expertise are available to handle medical emergencies during the study. Responsible physicians must also ensure that all personnel involved in the study are familiar with the contents of this protocol section.

Patient participation in the study will be noted in the patient record, which accompanies the patient during the hospital stay.

Definitions and reporting is strictly defined in Appendix B.

Patients were observed in the clinic during the 3-4 hours it takes to complete the invasive examination procedure. Emergency medical treatment is available

### Adverse events

Adverse Event in a clinical trial is an undesirable event when a drug is administered for purposes of study regardless of whether the incident is believed to be related to drug treatment or not. There may be a symptom, medical signs of disease, abnormally laboratory test or deterioration of an existing condition. In a clinical study, the adverse events should be recorded from the day that the person signed the informed consent to final visit, alternatively, some time after the last visit. The intensity (mild, moderate or severe), and severity (severe or non-severe) should be assessed. An adverse event should be followed up for as long as it is considered medically relevant

### Serious adverse events

SAEs (Serious Adverse Events) and discontinuations due to adverse events must be collected, and an assessment of causality of the SAE should be performed. An SAE is an event that at any dose results in one of the following

- Death
- A life-threatening event
- Hospital care or prolonged hospitalization
- A persistent or significant disability or incapacity
- A congenital anomaly or birth defect

An important medical event that may not result in death, but that could be life-threatening or require hospitalization may be considered an SAE when it is based on appropriate medical judgment.

Principal investigator of the current study is required to immediately report to the Ethics Committee and Regulatory Authority on an SAE occurs. A related assessment to be made.

The following terms and definitions used in the assessment of the causal relationship between each SAE and given the study drug:

• Clear - There is no doubt that the incident is related

• Probable - reasons and sufficient documentation to assume a causal relationship

• Possible - A causal relationship can not be excluded and can not be dismissed

• Unlikely - The event is most likely related to another aetiology than the study drug

• Unknown / unclassifiable: An adverse reaction which can not be assessed due to insufficient or

### Suspected Unexpected Serious Adverse Reactions

Sponsor who is also the principal investigator of the current study, is responsible for the reporting of serious adverse events that are unexpected with investigational medicinal products, known SUSARs (Suspected Unexpected Serious Adverse Reactions), to the EudraVigilance database and to the Ethics Committee. Fatal and life-threatening adverse events must be reported within 7 days after they have come to the investigator's knowledge. A detailed report must be received by another 8 days. For other SUSARs valid for 15 days. For the current study, the sponsor / investigator is responsible to delegate registration SUSARs in the EudraVigilance database to the MPA. Use CIOMS form and send it electronically to [registrator@mpa.se](mailto:registrator@mpa.se)

### Summary

An annual safety statement must be sent to the Medical Products Agency and the Ethics Review Board as long as the study is ongoing (see Schedule 9.6) where a summary is made of all SAEs occurred and SUSARs. Current reference safety information contained in FASS and SPC will apply.

Independent Security will not be used in this study.

Recording of adverse events will be made continuously during the study.

# STATISTICS

## Power calculation

Taking into account previous experience and current power calculations, we estimate that 30 individuals should be enough to reveal a biologically relevant difference (roughly 50%) in this experimental test set-up. The subjects are their own controls which significantly increases the power (probability of detecting a difference) since we avoid inter-individual variation.

## Analytical methods

Standard methods will be used for statistical analyzes of the study data. SPSS software program will be used for statistical calculation.

Values will be presented as means and SEM. Student's t-test will be used for comparisons between treatment groups. Comparisons in respons to Isoprenalin will be evaluated by two-way (treatment / no treatment and time) ANOVA for repeated measures. Chi-square test will be used for comparison of categorical data. Results will be considered statistically significant if P <0.05 (two-tailed tests).

# SAMPLE AND DATA MANAGEMENT STUDY

Results of clinical examination records are kept according to standard practice. The patients signed consents and other essential documents are saved in the study file. Study-specific examinations, dates of surveys and analysis results are recorded in individual CRFer

## Quality Control and Assurance

Study nurse Erika Backlund (who is not directly involved in the clinical management of patients) are monitor for the study. The monitor has undergone GCP training memory. Qualifications should be documented. She must be well informed in the study and its purpose, the content of patient information and to know the concept and content of the GCP, and national and international regulations related to clinical drug trials. The monitor shall have regular contact with the various study centers, check that there is a signed consent for all patients included, distribute vaccines, ensure that the study protocol is followed and that the data is correctly inserted in the patient records / CRF.

## Data Management

Collected data will be coded and stored on an external hard drive. Key for un-identification is kept in a locked cabinet at the Secretary of the laboratory and will be available only to those persons who are directly involved in the research project. Researchers have continuous access to unidentified raw data and the processed material via laptop related research at the Wallenberg Laboratory, Sahlgrenska University Hospital. Dr. Niklas Bergh is responsible for continuous data processing and report writing.

Details of the patient participating in a clinical study, the consent date, name of the study (VPL-02), and given the study drug (valproic acid) must be recorded, saved and stored according to clinical practice in the hospital's electronic medical record Melior.

A paper CRF is established for each patient. The CRF serves as source data regarding medical history, date of study days, blood sampling, adverse event registration and medication list. Blood flow recordings (CD), pletyhysmografic recordings, ECG and test results from the analysis lab represents the source data.

One main folder set up in which all the essential documents to be stored. Including signed consents shall be stored in the study binder.

All original data pertaining to the study, including signed consent form will be stored in files in a safe place during the implementation of the study and at least 10 years after the end of the study. Responsible study physician is responsible for the management and archiving of data.

### Analysis of data

Unidentified data will be processed in principle investigator password protected computer. Any evaluation of the collected data, including plethysmografic recordings and statistical analyzes, are blinded to the controller.

## Biological material

All biological samples will be handled and stored in accordance with the Biobank Act. The samples will be stored in the registered biobank available on Cardiology, Sahlgrenska University Hospital / SU, National Board number 143, Principal Lars Grip.

Blood plasma will be stored under the subjects' allocation numbers and stored in coded tubes in -70 degrees Celsius in the Wallenberg Laboratory, Sahlgrenska University Hospital, Bruna Stråket 16, 413 45 Gothenburg.

The subjects have given their written consent to the samples included in a biobank, and may at any time request that the samples be destroyed.

Materials that can be linked to personal information will not be disclosed to anyone outside the research team without the volunteers giving their consent for that use has been approved by the Research Ethics Board and the notification of the release of tissue samples from the biobank is made.

## Access to relevant security / staff

Principal investigator at each centre must ensure that the personnel involved receive adequate information and education and to eventual changes in the implementation of the study informed to. Clinical examination and sampling will be performed at the hospital's cardiothoracic department. These clinics are fully equipped with intensive care equipment. Survey and sampling will be performed by a specialist qualified doctor and nurse.

## Insurance

The subjects are covered by the Patient Injury Insurance Pharmaceutical Insurance as in health care in general.

## Timeplan

The study duration for each subject will be approximately 9-10 weeks from study day 1 to study day 4. The study is expected to be completed in approximately one year.

First patient first visit: February-March 2013 The last patient's last visit is expected to December 2014.

### Closed trial

The last visit of the last patient in the study is defined as the completion of trial.

## Reports and Publications

Sponsor will within 3 months after the trial in its entirety is completed at all study centres, report to the Medical Products Agency and the Ethics Committee to trial is ended as defined in Section 8.6.

A summary report will be sent to the MPA 1 year after the study as a whole is completed.

All of the scientists involved in the study will be part of the discovery made, and participate in the compilation of the research findings, which will be published in all national as well international scientific journals.

# References

1. Cardillo C, Kilcoyne C, Quyyumi A, Cannon and Panza J. Decreased vasodilator response to isoproterenol during nitric oxide inhibition in man. Hypertension 1997; 30: 918-921.
2. Chandler WL, Levy WC and Stratton JR. The circulatory regulation of TPA and UPA secretion, clearance, and inhibition during exercise and during the infusion of isoproterenol and phenylephrine. Circulation 1995;92:2984-2994.
3. Crompton G. A brief history of inhaled asthma therapy over the last fifty years. Primary Care Resp J 2006;15:326-331.
4. DeSouza CA, Clevenger CM, Greiner JJ, Smith DT, Hoetzer GL, Shapiro LF and Stauffer BL. Evidence for agonist-specific endothelial vasodilator dysfunction with ageing in healthy humans. J Physiol 2002;542:255-262.
5. Giannarelli C, Virdis A, De Negri F, Duranti E, Magagna A, Ghiadoni L, Salvetti A and Taddei S. Tissue-type plasminogen activator release in healthy subjects and hypertensive patients: relationship with beta-adrenergic receptors and the nitric oxide pathway. Hypertension 2008;52:314-321.
6. Hume KM and Gandevia B. Forced expiratory volume before and after isoprenaline. Thorax 1957;12:279.
7. Khalsa SS, Rudrauf D, Sandesara C, OlshandskyB and Tranel D. Bolus isoproterenol infusions provide a reliable method for assessing iteroceptive awareness. Int J Psychophysiol 2009;72(1):34-45.
8. MacLean LD, Duff JH, Scott HM and Peretz DI. Treatment of shock in man based on hemodynamic diagnosis. Surg Gynecol Obstet 1965;1120:1-16.
9. Nathanson MH and Miller H. The action of norepinephrine, epinephrine and isopropylnorepinephrine on the rhythmic function of the heart. Circulation 1952;6(2):238-44.
10. Reedwood D. Conservative treatment of chronic heart block. Brit med J 1969;1:26-29.
11. Santman FW. Catecholamines in critical care. The commonly used catecholamines: receptor and clinical profile, indications and dosages. Pharm Weekbl Sci 1992;14(5):290-296
12. Schindler C, Dobrev D, Grossmann M, Francke K, Pittrow D and Kirch W. Mechanisms of beta-adrenergic receptor-mediated venodilation in humans. Clin Pharmacol Ther 2004;75:49-59.
13. Schmetterer L, Sallinger S, Polak K, Eichler HG and Wolzt. NITRIC OXIDE. Biology and chemistry 1999;3(3):209-215.
14. Stein CM, Brown N, Vaughan DE, Lang CC, Wood AJ. Regulation of local tissue-type plasminogen activator release by endothelium-dependent and endothelium-independent agonists in human vasculature. J Am Coll Cardiol 1998;32:117-122.
15. Reinsfelt B, Ricksten SE, Zetterberg H, Blennow K, Fredén-Lindqvist J, Westerlind A. Cerebrospinal Fluid Markers of Brain Injury, Inflammation, and Blood-Brain Barrier Dysfunction in Cardiac Surgery. Ann Thorac Surg. 2012 Jun 12.

| **Clinical Study protocol Appendix A** | |
| --- | --- |
| Study code: | VPA-02 |
| EUdraCT nb: | 2012-004950-27 |
| Date: | 2012-11-15 |

| **Signatures of the persons responsible for the study**    I agree to the terms and conditions of this study protocol | | |
| --- | --- | --- |
|  | | |
|  |  |  |
|  |  | Date (year, month, day) |
|  |  |  |
|  |  |  |
|  |  | Date (year, month, day) |
|  |  |  |
|  |  |  |
|  |  | Date (year, month, day) |
|  |  |  |
|  |  |  |
|  |  | Date (year, month, day) |
|  |  |  |
|  |  |  |

| **Appendix B - Definitions of Adverse Events and Procedures in case of Pregnancy** | |
| --- | --- |
|  | |
| Definitions: |  |
| Adverse Events |  |
| Serious Adverse Events |  |
| A medical emergency usually constitutes an SAE and is to be reported as such |  |
| Intensity rating |  |
| Causal relationship |  |
| Action taken |  |
| Reporting in CRF |  |
| Adverse Events based on signs and symptoms |  |
| Final outcome assessment |  |
| Reporting of serious adverse events |  |
| Further guidance on Serious Adverse Events: |  |
| Life threatening |  |
| Hospitalisation |  |
| Important medical event or medical intervention |  |
| A guide to interpreting the causality QUESTION |  |
| Other significant Adverse Events |  |
| Procedures in case of pregnancy |  |
| **Maternal exposure** |  |
| **Overdose** |  |

DEFINITIONS OF ADVERSE EVENTS AND PROCEDURES IN CASE OF PREGNANCY

It is of the utmost importance that all staff involved in the study is familiar with the content of this section. The Principal Investigator is responsible for ensuring this.

Adverse Events

An adverse event is the development of an undesirable medical condition or the deterioration of a pre-existing medical condition following or during exposure to a pharmaceutical product, whether or not considered causally related to the product. An undesirable medical condition can be symptoms (eg, nausea, chest pain), signs (eg, tachycardia, enlarged liver) or the abnormal results of an investigation (eg, laboratory findings, electrocardiogram). In clinical studies, an AE can include an undesirable medical condition occurring at any time, including run-in or washout periods, even if no study treatment has been administered.

The term AE is used to include both serious and non-serious AEs

Serious Adverse Events and suspected unexpected serious adverse reactions (SUSAR)

It is important to distinguish between Serious Adverse Events (SAEs) and severe adverse events (AEs). Severity is a measure of intensity whereas seriousness is defined by the criteria listed below. An AE of severe intensity need not necessarily be considered serious. For example, nausea which persists for several hours may be considered severe nausea, but not an SAE. On the other hand, a stroke which results in only a limited degree of disability may be considered a mild stroke but would be a SAE.

**A medical emergency usually constitutes an SAE and is to be reported as such**

A serious adverse event is an AE occurring during any study phase (i.e. run-in, pre-entry, screening, treatment, wash-out, follow-up), and at any dose of the investigational product, comparator or placebo, that fulfils one or more of the following criteria:

- results in death
- is immediately life-threatening
- requires in-patient hospitalisation or prolongation of existing hospitalisation
- results in persistent or significant disability or incapacity
- is a congenital abnormality or birth defect
- is an important medical event that may jeopardise the subject or may require medical intervention to prevent one of the outcomes listed above.

For reporting purposes, any suspected transmission via a medicinal product of an infectious agent is also considered an SAE and is reported in an expedited manner. Any organism, virus or infectious particle (for example prion protein transmitting Transmissible Spongiform Encephalopathy), pathogenic or non-pathogenic, is considered an infectious agent.

**Definitions for severity rating**

1. None – No symptoms
2. Mild - Transient symptoms; awareness of sign or symptom, but easily tolerated. No interference with the subject’s daily activities
3. Moderate - Marked symptoms; discomfort sufficient to cause interference with normal activities. Moderate interference with the subject’s daily activities
4. Severe - Considerable interference with the subject’s daily activities; unacceptable, incapacitating. Inability to perform normal activities

N/A Not Applicable

**Causal relationship**

The causality of (S)AEs (ie, their relationship to study treatment and/or the investigational procedure) will be assessed by the investigator(s), who in completing the relevant case report form must answer “yes” or “no” to the question “Do you consider that there is a reasonable possibility that the event may have been caused by the drug/the investigational procedure?”

The following terms and definitions are used when assessing the causal relationship between each AE and the relevant trial product(s):

1. Definite - There is no doubt that the incident is related
2. Probable - Good reason and sufficient documentation to assume a causal relationship
3. Possible- A causal relationship is conceivable and cannot be dismissed
4. Unlikely - The event is most likely related to aetiology other than the trial product
5. Not related – The event is not related to the trail product
6. Unknown/Unclassifiable: a report suggesting an adverse event reaction, which cannot be judged because information is insufficient or contradictory and which cannot be supplemented or verified.

N/A Not Applicable

Causal relationship in cases where the disease under study has deteriorated due to lack of effect will be classified as no reasonable possibility.

For SAEs causal relationship will also be assessed for additional study drug and/or other medication and/or study procedure. Note that for SAEs that could be associated with any study procedure the causal relationship is implied as “yes”.

**Action taken**:

1. None
2. Dose of study drug changed
3. Study drug temporarily stopped
4. Study drug stopped

N/A Not Applicable

**Reporting in the Case report Form**

The following variables will be recorded in the CRF for each AE; description of the AE, the date and time when the AE started and stopped, maximum intensity*,* whether the AE is serious or not, causality rating (yes or no; if yes specify), action taken with regard to investigational product, AE caused subject to discontinue study and outcome.

Adverse Events based on signs and symptoms

All AEs spontaneously reported by the subject or reported in response to the open question from the study personnel: *“Have you had any health problems since the previous visit?”*, or revealed by observation will be collected and recorded in the CRF. When collecting AEs the recording of diagnoses is preferred (when possible) to recording a list of signs and symptoms. However, if a diagnosis is known and there are other signs or symptoms that are not generally part of the diagnosis, the diagnosis and each sign or symptom will be recorded separately.

Follow-up – Outcome assessment

Any AEs that are unresolved at the patient’s last AE assessment in the study are followed up by the investigator for as long as medically indicated, but without further recording in the CRF.

The following terms and definitions are used in assessing the final outcome of an AE:

- Recovered - The subject has fully recovered, or by medical or surgical treatment the condition has returned to the level observed at the first trial-related activity after the subject signed the informed consent.
- Recovering - This term is only applicable if the subject has completed the trial or has died from another AE. The condition is improving and the subject is expected to recover from the event.
- Recovered with sequelae - The subject has recovered from the condition, but with lasting effect due to a disease, injury, treatment or procedure. If a sequela meets an SAE criterion, the AE must be reported as an SAE.
- Not recovered - The condition of the subject has not improved and the symptoms are unchanged, or the outcome is not known at the time of reporting.
- Fatal - This term is only applicable if the subject died from a condition related to the reported AE. Outcomes of other reported AEs in a subject before he/she died should be assessed as “recovered”, “recovering”, “recovered with sequelae” or “not recovered”. An AE with fatal outcome must be reported as an SAE.
- Unknown - This term is only applicable if the subject is lost to follow-up

**Reporting of serious adverse events**

For studies in countries implementing the EU Clinical Trials Directive, informing Ethics Committees and Regulatory Authorities will be performed by the sponsor.

All Suspected Unexpected Serious Adverse Reactions (SUSARs) have to be reported, whether or not considered causally related to the investigational product, or to the study procedure(s). The Clinical Study Serious Adverse Event Report Form will be used together with other relevant supporting documentation (e.g. ECG, laboratory results, autopsy report) and relevant CRF modules. All SUSARs have to be electronically registered in the EMEAs database.

**FURTHER GUIDELINES ON THE DEFINITION OF A SERIOUS ADVERSE EVENT**

Life threatening

‘Life-threatening’ means that the subject was at immediate risk of death from the AE as it occurred or it is suspected that use or continued use of the product would result in the subject’s death. ‘Life-threatening’ does not mean that had an AE occurred in a more severe form it might have caused death (eg, hepatitis that resolved without hepatic failure).

Hospitalisation

Out-patient treatment in an emergency room is not in itself a serious AE, although the reasons for it may be (eg, bronchospasm, laryngeal oedema). Hospital admissions and/or surgical operations planned before or during a study are not considered AEs if the illness or disease existed before the subject was enrolled in the study, provided that it did not deteriorate in an unexpected way during the study.

Important medical event or medical intervention

Medical and scientific judgement should be exercised in deciding whether a case is serious in situations where important medical events may not be immediately life-threatening or result in death, hospitalisation, disability or incapacity but may jeopardize the subject or may require medical intervention to prevent one or more outcomes listed in the definition of serious. These should usually be considered as serious.

*Examples of such events are:*

*- Angioedema not severe enough to require intubation but requiring iv hydrocortisone treatment*

*- Hepatotoxicity caused by paracetamol (acetaminophen) overdose requiring treatment with N-acetylcysteine*

*- Intensive treatment in an emergency room or at home for allergic bronchospasm*

*- Blood dyscrasias (eg, neutropenia or anaemia requiring blood transfusion, etc) or convulsions that do not result in hospitalisation*

*- Development of drug dependency or drug abuse*

A guide to interpreting the causality QUESTION
The following factors should be considered when deciding if there is a “reasonable possibility” that an AE may have been caused by the drug.

- Time Course. Exposure to suspect drug. Has the subject actually received the suspect drug? Did the AE occur in a reasonable temporal relationship to the administration of the suspect drug?
- Consistency with known drug profile. Was the AE consistent with the previous knowledge of the suspect drug (pharmacology and toxicology) or drugs of the same pharmacological class? OR could the AE be anticipated from its pharmacological properties?
- Dechallenge experience. Did the AE resolve or improve on stopping or reducing the dose of the suspect drug?
- No alternative cause. The AE cannot be reasonably explained by aetiology such as the underlying disease, other drugs, other host or environmental factors.
- Rechallenge experience. Did the AE reoccur if the suspected drug was reintroduced after having been stopped? A re-challenge would not normally be recommended or supported.
- Laboratory tests. A specific laboratory investigation (if performed) has confirmed the relationship?

A “reasonable possibility” could be considered to exist for an AE where one or more of these factors exist.

In contrast, there would not be a “reasonable possibility” of causality if none of the above criteria apply or where there is evidence of exposure and a reasonable time course but any dechallenge (if performed) is negative or ambiguous or there is another more likely cause of the AE.

In difficult cases, other factors could be considered such as:

- Is this a recognised feature of overdose of the drug?
- Is there a known mechanism?

Ambiguous cases should be considered as being a “reasonable possibility” of a causal relationship unless further evidence becomes available to refute this. Causal relationship in cases where the disease under study has deteriorated due to lack of effect should be classified as no reasonable possibility.

Other significant Adverse Events

An expert will identify other significant Adverse Events (OAEs) during the evaluation of safety data for the Clinical Study Report. Significant adverse events of particular clinical importance, other than SAEs and those AEs leading to discontinuation of the subject from study treatment, will be classified as OAEs. Examples of these are marked haematological and other laboratory abnormalities, and certain events that lead to intervention (other than those already classified as serious), dose reduction or significant additional treatment. For each OAE, a narrative will be written and included in the Clinical Study Report.

Procedures in case of pregnancy

Pregnancy itself is not regarded as an adverse event unless there is a suspicion that the investigational product under study may have interfered with the effectiveness of a contraceptive medication. The outcome of all pregnancies (spontaneous miscarriage, elective termination, normal birth or congenital abnormality) must be followed up and documented even if the subject was discontinued from the study. All reports of congenital abnormalities/birth defects are SAEs. Spontaneous miscarriages should also be reported and handled as SAEs. Elective abortions without complications should not be handled as AEs. All outcomes of pregnancy must be reported to PI on the pregnancy outcomes report form.

Part I of this form must be completed in full and returned to PI within 30 days. Part II of the form must be completed when the outcome of the pregnancy is known. Reports of normal outcomes should be sent within 30 days.

**Maternal exposure**

Pregnancy itself is not regarded as an adverse event unless there is a suspicion that the investigational product under study may have interfered with the effectiveness of a contraceptive medication. Congenital abnormalities/birth defects and spontaneous miscarriages should be reported and handled as SAEs. Elective abortions without complications should not be handled as AEs. The outcome of all pregnancies (spontaneous miscarriage, elective termination, normal birth or congenital abnormality) must be followed up and documented even if the subject was discontinued from the study.

**Overdose**

- An overdose with associated AEs is recorded as the AE diagnosis/symptoms on the relevant AE modules in the CRF.

| **Clinical study-protocol Appendix C** | |
| --- | --- |
| Study code: | VPA-02 |
| EUdraCT nb: | 2012-004950-27 |
| Date: | 2012-10-23 |

**Protocol for invasive investigation**

**Before the test:**

1 Forearm Volume is measured

2 control of checklist

3 Venflon in the dominant antecubital

4 Blood tests: fB-glucose, lipids (study day I), S-valproate (study day I and II)

5 catheterization non-dominant brachial artery according to SOP

6 Venflon in the non-dominant antecubital

7 ECG and ia pressure linked

8 plethysmography connected according to SOP

9 Rest, about 30 min after catheterization

10 trials are carried out according to protocols

**Sampling Schedule at invasive test:**

**Baseline 1: 15 min:** Blood**-**Sampling at 5 and 10 min, **2** **ml art and vein**

**Provocation with Isoprenaline 200 ng/min during 20 min:**

Blood-sampling 2 ml **vein** at 1.5, 3, 6, 9, 12, 15, and 18 minutes and **2 ml art** at 20 minutes.

**Baseline 2:** Blood**-**Sampling at 0 min: **2** **ml art and vein**

**Provocation with Isoprenaline 200 ng/min during 20 min:**

Blood-sampling 2 ml **vein** at 1.5, 3, 6, 9, 12, 15, and 18 minutes and **2 ml art** at 20 minutes.

**Baseline 2:** Blood**-**Sampling at 0 min: **2** **ml art and vein**

At all timepoints 2ml slush is drawn before blood-sampling. 2 ml Stabilyte® 1/10 in tubes. Flush 2 mL with NaCl 0.9%. Plethysmography, MAP and HR are recorded immediately after the blood sample is drawn

**Upon completion forearm attempt**

1. ECG / plethysmography disconnected. Venflon drawn. Artery Catheter pulled, ≥20 my compression of the puncture site, as per SOP.
2. The subject gets brought home information about the procedure and possible. measures in case of complications
3. Catheterisation protocol be filled

**If side-effects**

1. Adverse effects of catheterisation are reported in the protocol. If more severe complications the principle investigator is informed who will consider action.
2. Adverse drug events in relevant occasions are reported in conventional manner to the MPA.
